# Nanoscale Ultrafine Zinc Metal Anodes for High Stability Aqueous Zinc Ion Batteries

**DOI:** 10.1021/acs.nanolett.2c03919

**Published:** 2023-01-03

**Authors:** Mingqiang Liu, Lu Yao, Yuchen Ji, Mingzheng Zhang, Yihang Gan, Yulu Cai, Hongyang Li, Wenguang Zhao, Yan Zhao, Zexin Zou, Runzhi Qin, Yuetao Wang, Lele Liu, Hao Liu, Kai Yang, Thomas S. Miller, Feng Pan, Jinlong Yang

**Affiliations:** † Guangdong Research Center for Interfacial Engineering of Functional Materials, College of Materials Science and Engineering, Shenzhen University, Shenzhen518060, P. R. China; ‡ School of Advanced Materials, Peking University Shenzhen Graduate School, Shenzhen518055, P. R. China; § Electrochemical Innovation Lab, Department of Chemical Engineering, University College London, London, WC1E 7JE, U.K.; ∥ Department of Mechanical Engineering, Imperial College London, London, SW7 2AZ, U.K.; ⊥ School of Chemical Engineering and Advanced Materials, The University of Adelaide, North Terrace, South Australia5005, Australia; # Department of Electrical and Electronic Engineering, University of Surrey, Guildford, SurreyGU2 7XH, U.K.

**Keywords:** Aqueous Zn batteries, zinc metal anode, ultrafine nanograins, dendrite growth, parasitic reactions

## Abstract

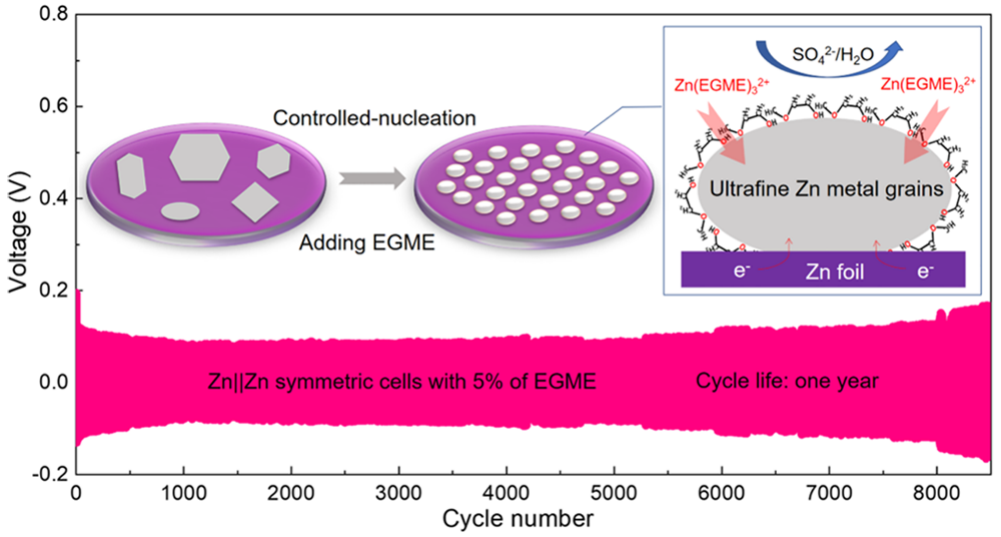

Aqueous Zn batteries (AZBs) are a promising energy storage technology, due to their high theoretical capacity, low redox potential, and safety. However, dendrite growth and parasitic reactions occurring at the surface of metallic Zn result in severe instability. Here we report a new method to achieve ultrafine Zn nanograin anodes by using ethylene glycol monomethyl ether (EGME) molecules to manipulate zinc nucleation and growth processes. It is demonstrated that EGME complexes with Zn^2+^ to moderately increase the driving force for nucleation, as well as adsorbs on the Zn surface to prevent H-corrosion and dendritic protuberances by refining the grains. As a result, the nanoscale anode delivers high Coulombic efficiency (ca. 99.5%), long-term cycle life (over 366 days and 8800 cycles), and outstanding compatibility with state-of-the-art cathodes (ZnVO and AC) in full cells. This work offers a new route for interfacial engineering in aqueous metal-ion batteries, with significant implications for the commercial future of AZBs.

In order to address the energy crisis and environmental pollution, a major focus of human endeavor in recent decades has been the development of electrochemical energy storage and conversion systems to exploit renewable energy sources and achieve the goal of carbon neutrality.^1−3^ Aqueous zinc batteries (AZBs) are widely considered to be much safer and cheaper alternatives to lithium-ion batteries (LIBs), due to their high theoretical capacities (820 mAh g^–1^) and the fact that they can operate in nonflammable and non-toxic aqueous electrolytes.^1,4−8^ However, the plating-stripping electrochemistry of zinc anodes in aqueous electrolytes has to date suffered from a low Coulombic efficiency (CE), caused by hydrogen evolution reaction (HER) corrosion side reactions associated with irreversible byproducts (Figure S1a).^9,10^ Large and disruptive hexagonal dendrites are easily formed in many AZBs, due to uneven deposition at the Zn metal surface, leading to capacity fading, consumption of electrolyte, and short circuits.^11,12^

Many methods have been tried to obtain a homogeneous surface and improve the electrochemical performance of Zn metal anodes, such as modulating zinc electrodeposition behavior,^13,14,12,11,15,16^ surface modification,^17−21^ 3D structural anodes,^22−25^ novel separators,^26,27^ electrolyte additives (including salts,^28−32^ organic molecules^2,33−38^), and more.^39^ Electrolyte optimization has also been widely researched as one of the most easy-to-implement and industrially relevant solutions, regulating the Zn^2+^ solvation sheath and introducing a stable solid electrolyte interphase, thereby inhibiting dendrite growth and the decomposition of H_2_O.^36,40−42^

Yet while, many of the above solutions have been proven to be effective in suppressing Zn dendrites growth or minimizing the HER and the formation of byproducts; few studies have focused on the issue of interfacial tension of electrolytes on metallic Zn surface, neglecting this crucial fundamental science. This is important as many of the problems associated with AZBs are exacerbated by the higher reactivity of metallic Zn in water-based electrolytes (−0.762 V vs standard hydrogen electrode (SHE)).^28,36^ According to the Nernst equation (Δ*G*_m_ = −*nFE*^θ^),^43^ the Gibbs free-energy (Δ*G*_m_ = −147 kJ mol^–1^) of the reaction (Zn + 2H^+^ = Zn^2+^ + H_2_) is < 0, meaning the spontaneity of Zn metal corrosion in zinc salt solutions^44^ triggers a series of side reactions, including HER and inert byproducts (eqs s1 and s2 in Figure S1a). Our prior work^16,45^ explored the use of a passivation layer to alter electrolyte surface tension, impede proton induced HER and protect the Zn metal from corrosion. But a simple and scalable method to controllably modulate electrolyte surface free energy and inhibit the activity of metallic zinc, thereby reducing side reactions and promoting uniform zinc deposition, is still required.

Herein, we propose a unique route to fabricate ultrafine grainy dendrite-free zinc metal anodes by decreasing the electrolyte surface tension and passivating the metallic Zn. Specifically, an organic molecule, ethylene glycol monomethyl ether (EGME), was introduced into a traditional ZnSO_4_ electrolyte, which acts to both coordinate with Zn^2+^ ions to modulate the solvation structure and to chemisorb to the metallic Zn surface, thereby controllably increasing the driving force for zinc nucleation and growth, promoting uniform deposition and preventing side reactions (eq s3 in Figure S1b). It is demonstrated that the refined zinc anodes can realize a high average CE of 99.5% and promote ultralong-term cycling stability over 8800 cycles (366 days). AC (activated carbon)||Zn full cells are also shown to display excellent cyclability, with negligible capacity fading over 10000 cycles, and larger pouch cells maintain near 100% retention over 1000 cycles, while full ZnVO||Zn cells demonstrate that EGME is compatible with state-of the-art ZIB cathodes. This simple method for controlling zinc anode interfacial structure and increasing AZB stability offers both a promising direction for understanding the mechanisms of zinc nucleation and growth in applied battery systems and a route for practically managing the anode instability issues that hold back the commercialization of AZBs and other energy storage systems.

## Theoretical Studies

**Figure 1:**
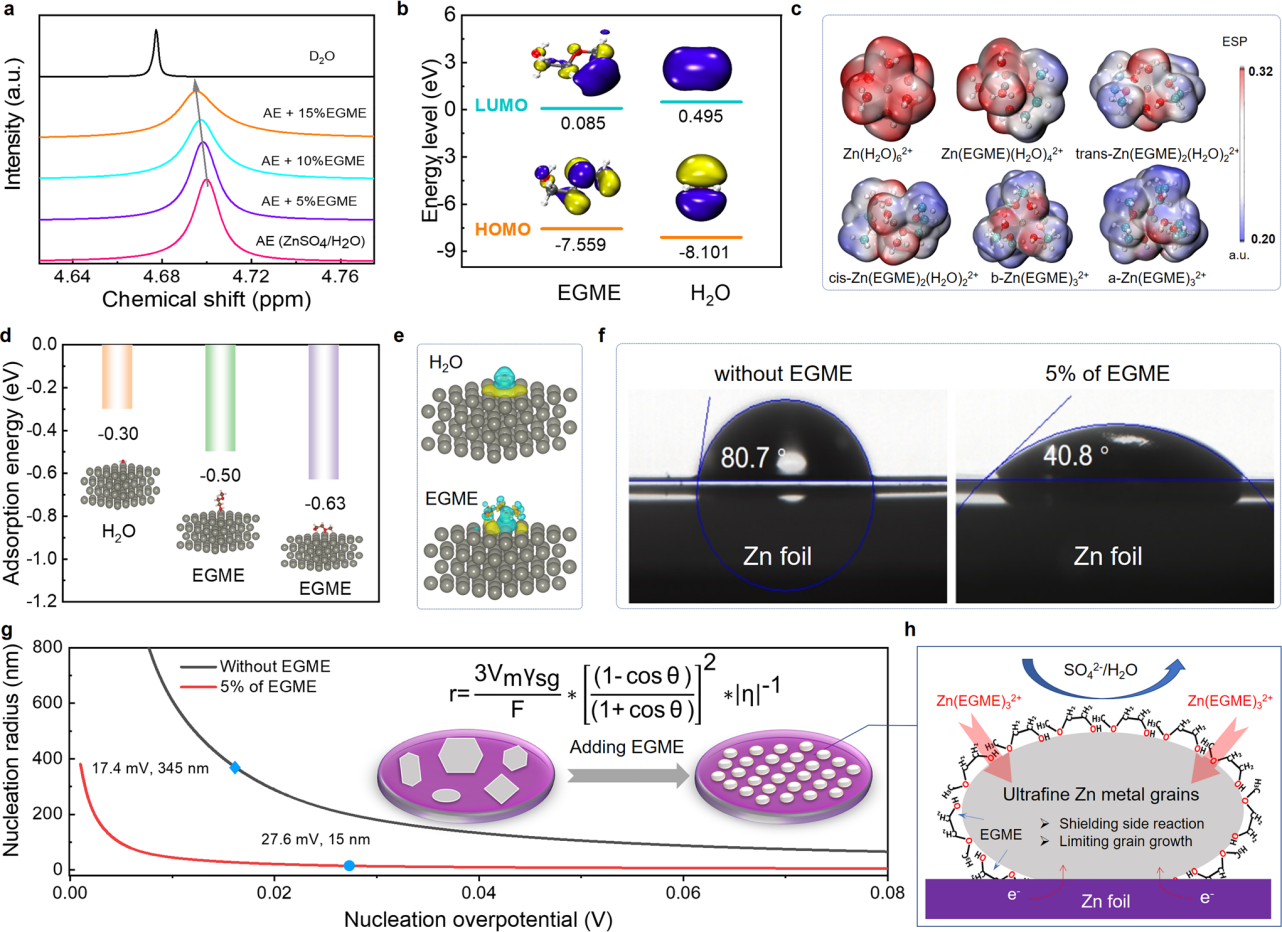
Theoretical studies. (a) ^1^H NMR spectra of a 3 M ZnSO_4_ aqueous electrolyte with different percentages of EGME added, from 0% to 15% in D_2_O. (b) LUMO and HOMO isosurfaces of EGME (left) and water molecules (right), (isovalue = 0.02 au). (c) Electrostatic potential mapping of Zn^2+^ with H_2_O and EGME molecule solvation structures. (d) Adsorption energy comparison of H_2_O and EGME molecules on a Zn (002) crystal plane with different modes of adsorption. (e) Corresponding absorbed models for the different modes. The charge density difference between H_2_O and EGME molecules adsorbed parallelly on Zn (002) crystal plane (yellow and light blue electron cloud clusters represent increase and decrease of electron density, respectively). (f) Wetting angles of ZnSO_4_ electrolytes without/with EGME on Zn foil. (g) Graph of the relationship between critical zinc nuclei radius and zinc nucleation overpotential. Insets are zinc nuclei, illustrating the differences in size and density of deposited zinc nuclei under varying electrolyte interfacial tension. (h) Schematic diagram of zinc ion deposition process in electrolytes with EGEM.

Theoretical calculations in scales ranging from atomistic-level quantum chemistry to molecule-level ab initio calculations were combined to investigate the deposition–dissolution process of zinc ions in AZBs. First, we investigated the effect of EGME molecules on the solvation structure of zinc ions. According to DFT calculations, the formation energy (Figure S2) of Zn^2+^ coordinated with EGME is 0.48–1.52 eV lower than that of Zn^2+^ coordinated with H_2_O molecules (−19.96 eV), meaning EGME can replace H_2_O in the solvation shell to obtain a stable solvated structure. Experimental NMR measurements of EGME in ZnSO_4_ electrolytes (Figure 1a and Figures S3 and S4) show peak movement to lower chemical shifts with the addition of EGME, which further demonstrate that EGME exhibits a strong tendency to complex with Zn^2+^ and displace water molecules from the typical solvation shell of [Zn(H_2_O)_6_]^2+^.

EGME molecules were also found to offer lower lowest unoccupied molecular orbital (LUMO) and higher highest occupied molecular orbital (HOMO) positions (Figure 1b) when compared to H_2_O (0.085 eV vs 0.495 eV; −7.559 eV vs −8.101 eV), implying that EGME can more easily lose electrons. While comparison of the electrostatic potentials (ESP) (Figure 1c and Figure S5) further showed that replacing H_2_O molecules in [Zn(H_2_O)_6_]^2+^ with EGME molecules is favorable.^46^ This behavior is consistent with the altered electrochemical behavior observed from 2D diffusion in the traditional aqueous electrolyte to 3D diffusion in the EGME containing electrolyte (detailed analysis can be found in the SI and Figure S7a). Thus, it was demonstrated that the EGME molecule can facilitate the deposition–dissolution process of zinc ions in AZBs.

The interactions between a Zn foil and the different molecules was studied via Multiwfn software. The corresponding adsorption energy (Figure 1d) of H_2_O and EGME via one or two O atoms on Zn (002) lattice plane were obtained to be −0.3, −0.5, and −0.63 eV, respectively, implying that strong binding via both EGME O sites to the Zn plane is preferential. Similar results were found when considering differences in charge density (Figure 1e). EGME was also found to adsorb more strongly than H_2_O at a number of Zn crystallographic planes (Figure S6), which may passivate the zinc metal to some extent and impact the nucleation–growth of zinc crystals.

Next, we explored the effect of EGME molecules on the zinc metal structure based on the nucleation–growth theory of electrodeposition.^47−49^ Voltage–time curves (Figure S7b) show two important characteristic overpotentials observed during galvanostatic Zn electrodeposition: (1) the nucleation overpotential (η_n_), which is the magnitude of the voltage spike at the onset of Zn deposition, and (2) the plateau overpotential (η_p_) present after nucleation occurs and Zn growth continues. The initial nucleation stage has a significant impact on the subsequent growth process as well as the structure and properties of the deposited products. Classical equations for homogeneous nucleation can be used to understand the dependence of the size of Zn nuclei on the electrodeposition overpotential and electrode and electrolyte properties. The nucleation radius (*r*) for forming a spherical nuclei is as follows:^50,47^
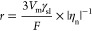
1Here *r* stands for the radius of zinc nuclei, *V*_m_ is the molar volume of zinc, *F* is Faraday’s constant, γ_sl_ is the surface energy of the Zn-electrolyte interface, which can be calculated according to the combination of Young’s equation^51^ and Fowkes equation^52^ and as follows:
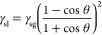
2Here γ_sg_ is the surface tension of the electrode and θ is the contact angle between the electrode and electrolyte, as shown in Figure 1f. Since the electrode is a fixed invariant, the nucleation radius (*r*) is influenced mainly by η_n_ and the contact angle (θ) determined by the nature of the electrolyte, the derived equation is as follows:
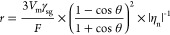
3

It can be clearly observed that the size of Zn crystals can be effectively refined by directly manipulating the electrolyte to reduce the wetting angle of the electrolyte on the substrate and increased η_n_. Interestingly, the contact angle of the electrolyte was greatly decreased to 40.8° after the addition of EGME, compared to 80.7° for the original ZnSO_4_ (Figure 1f), indicating a greater surface free energy of the EGME solvent than water on zinc foils (Figure S18). The corresponding nucleus radius–overpotential curves (eq 3) at the two wetting angles are presented in Figure 1g, where it can be seen that all of the zinc nuclei in the EGME-added electrolyte had radii that were consistently below those in the ZnSO_4_ electrolyte. Typically, an overpotential of 27.6 mV at a current density of 0.5 mA cm^–2^ in the traditional electrolyte corresponds to a nucleation radius of 345 nm, whereas an overpotential of 17.4 mV in an EGME-added electrolyte at the same current density corresponds to a nucleation radius of 15 nm, demonstrating that EGME molecules can achieve grain refinement through controlled nucleation. Therefore, this demonstrates that the EGME molecules with their optimal dioxygen functional groups replace H_2_O molecules in complex with Zn ions to increase the driving force for nucleation, as well as adsorbing on the metallic Zn surface and act as a passivation layer to prevent corrosion, regulating Zn deposition at kinetically favored sites, refining grains, promoting even growth and blocking dendritic protuberances (Figure 1h).

## Structural Evolution of Zinc Anodes

**Figure 2:**
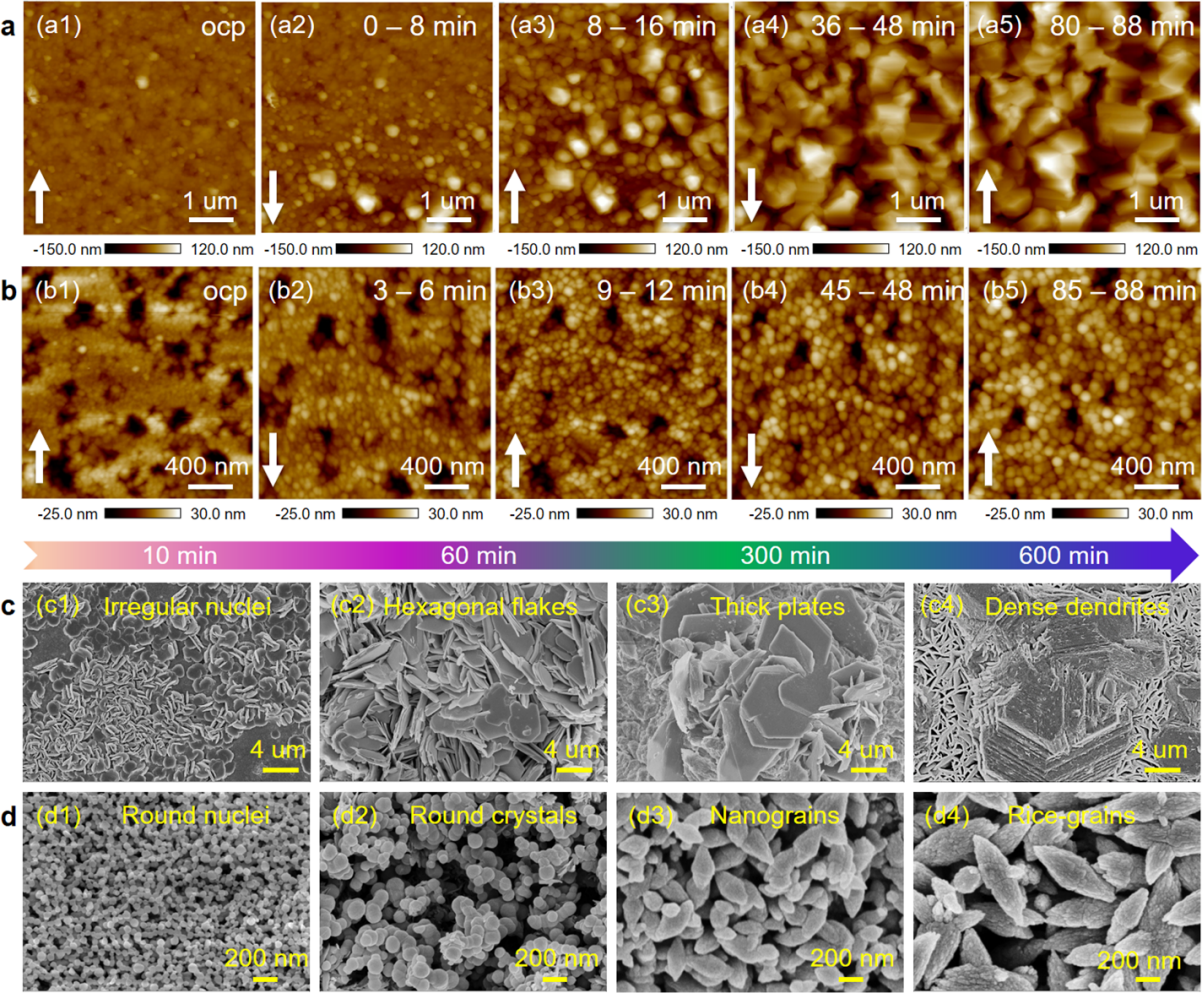
Structural evolution of the zinc anode. (a, b) In situ AFM images of zinc nucleation and growth process and (c, d) SEM morphology of Zn electrodeposits at different times (10, 60, 300, 600 min) (a, c) in ZnSO_4_ electrolytes and (b, d) 5% of EGME added ZnSO_4_ electrolytes.

The morphology of zinc nuclei and subsequent crystal growth under the regulation of EGME was uncovered by operando electrochemical atomic force microscopy (EC-AFM, set up shown in Figure S8), combined with ex situ scanning electron microscopy (SEM) and transmission electron microscopy (TEM). All operando EC-AFM images of galvanostatic Zn electrodeposition on Cu foil between 0 and 88 min at 0.1 mA cm^–2^ are shown in Figure S9 and Figure S10, with select zoomed sections highlighted in Figure 2, parts a and b. In original electrolyte small irregular-shaped zinc crystals can be seen to be produced at the early stages of electrodeposition and some of them gradually grow into large quasi-hexagonal zinc grains with edges and corners (Figure 2a2). After 88 min, the size of the zinc protrusions has reached 1–2 μm (Figure 2a5). In contrast, with the addition of EGME, the early zinc crystals are much smaller and more regular round particles (Figure 2b2). Thereafter, zinc nanograins grow slightly into spherical pellets that are tightly packed together, eventually forming a homogeneous surface with zinc nanoparticles of ∼100 nm (Figure 2b5). 3D AFM images (Figure S11) and corresponding surface roughness measurements (Figure S12) highlight the significant difference in Zn deposition morphology. The quantified surface roughness value (Ra) of the Zn on Cu in pure ZnSO_4_ increases dramatically from 9.4 at open circuit potential (OCP) to 20.4, 33.5, 39.5, and 47.5 from 0 to 8, 8 to 16, 36 to 48 and 80 to 88 min respectively, whereas the sample in the EGME-modified electrolyte remained almost unchanged at ∼8.0 Ra (Figure S13).

SEM images further show the differing microstructure of zinc electrodeposits between 10 and 600 min (Figure 2, parts c and d) (detailed analysis can be found in Figures S14–S17). The average size of Zn flakes after 300 min of deposition was 7.0 μm (Figure S16a–c), a size that would certainly cause damage within an operational AZBs. However, by regulating deposition using EGME, the zinc crystals grew into prolate spheroid (i.e., rice-shaped) nanopellets of ∼250 nm (Figure S16d–f), and then self-assembled into bigger nanocrystals of ∼400 nm after 600 min (Figure 2d4).

**Figure 3:**
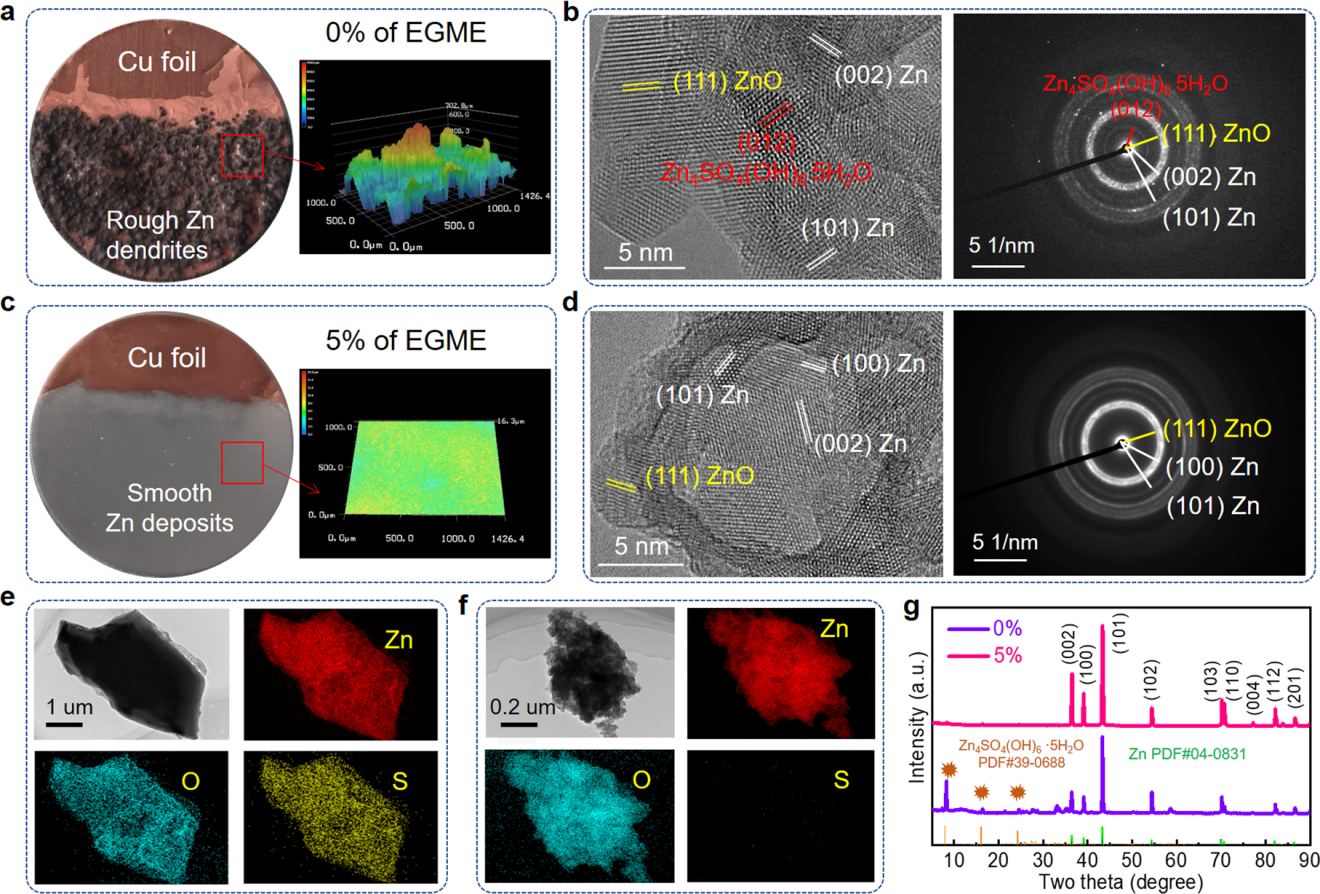
Structural characterization of zinc anodes. (a, b) Digital photos and corresponding 3D LCSM images of a Cu foil after electroplating, (c, d) HRTEM images and corresponding FFT patterns, (e, f) EDS maps of Zn, O, and S elements for zinc deposits at the Cu surface, and (g) XRD patterns of Zn foils after cycling ten times in (a, c, e, g) ZnSO_4_ electrolytes and (b, d, f, g) 5% of EGME added ZnSO_4_electrolytes.

Macroscale images of zinc electrodeposits on copper foil surfaces in electrolytes without/with EGME are shown in Figure 3, highlighting a stark morphological contrast. It can be clearly observed from Figure 3a that the surface of the Cu foil after plating in ZnSO_4_ electrolytes is black-brown and very rough. 3D laser confocal scanning microscope (LCSM) shows a surface roughness of over 700 μm. Interestingly, TEM imaging and electron diffraction data (Figure 3b) confirm the presence of Zn_4_SO_4_(OH)_6_·5H_2_O byproducts in the pure ZnSO_4_ cycled electrode, which has been linked to poor cycling performance and dendrite proliferation,^53^ alongside ZnO (suggesting oxidization after air exposure). In contrast, with the addition of EGME, the macroscale surface of the Zn coated Cu foil is extremely smooth with a zinc–metallic luster, consistent with the micro/nanoscale data (Figure 3c). The LCSM measured surface roughness was dramatically reduced to just 16.3 μm, over 43 times lower, highlighting grain refining effect of EGME molecules. Meanwhile, no detrimental Zn_4_SO_4_(OH)_6_·5H_2_O could be found from the surface of a single spherical particle under TEM (Figure 3d), only contributions from ZnO. In addition, energy dispersive X-ray spectroscopy (EDS) mapping from both SEM (Figure S18a and Table S1) and TEM (Figure 3e and Table S2) shows that the electrode cycled in pure ZnSO_4_ presents a significant S contribution (6.5 wt % TEM-EDS, 4.9 wt % SEM-EDS), implying large amounts of SO_4_^2–^ derived species have been formed during electrodeposition. However, little S was detected at the surface of the electrode cycled in EGME (0.08 wt % TEM-EDS, Figure 3f and Table S2, and 0.09 wt % SEM-EDS, Figure S18 and Table S1). The morphology of Zn foil electrodes after stripping for 600 min shows similar behavior (Figure S19), implying a strong contrast in heterogeneous and homogeneous reactive sites. X-ray diffraction patterns (XRD) and X-ray photoelectron spectroscopy (XPS) (Figure 3g and Figures S20–S22) of the cycled Zn anode with EGME further demonstrated that minimal amounts of byproducts were produced, showing that EGME has a significant ability to minimize parasitic reactions during cycling (detailed analysis can be found in the Supporting Information).

## Zinc Plating/Stripping Behaviors

**Figure 4:**
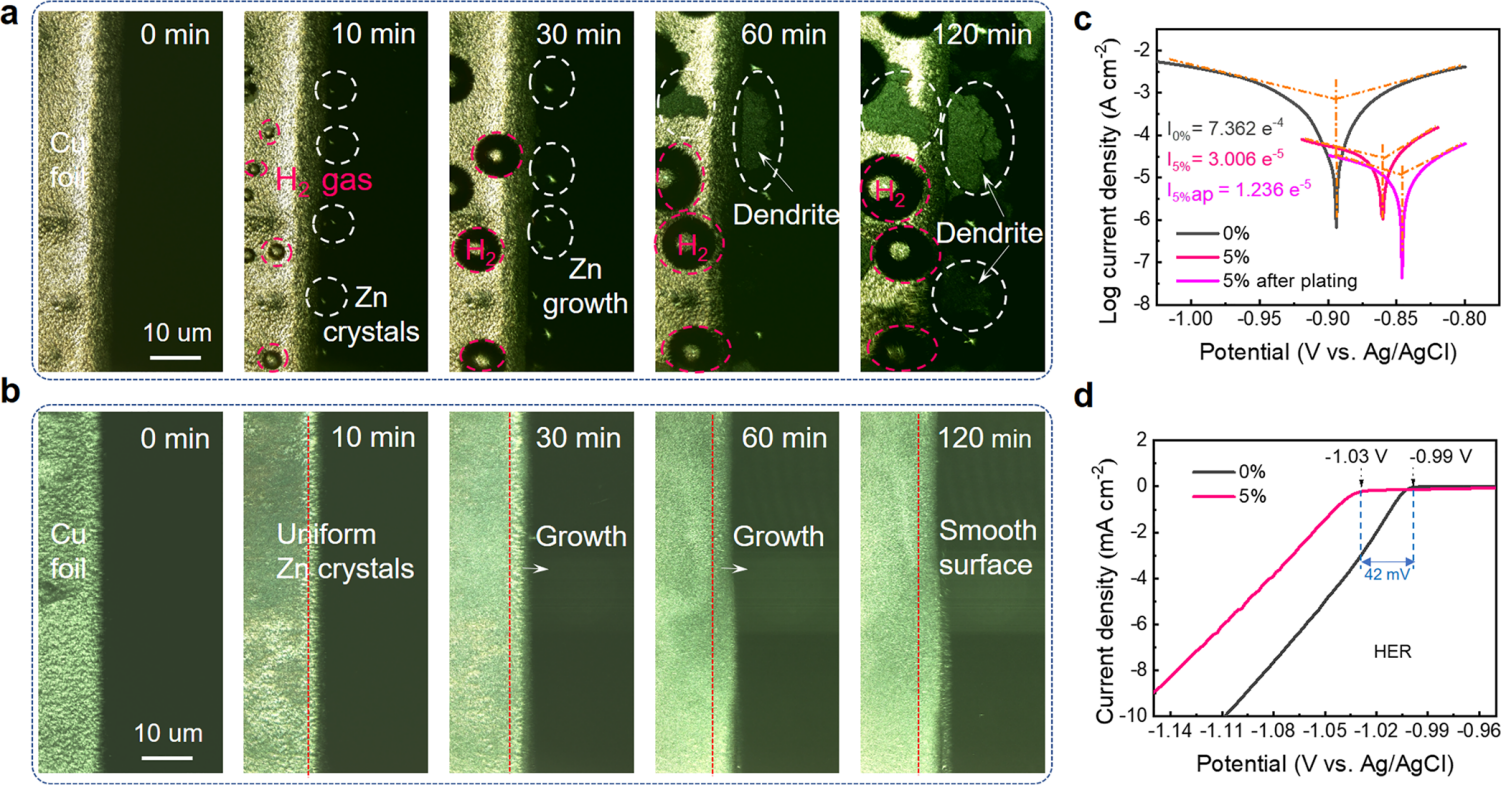
**Zinc Plating/Stripping Behaviors**. (a, b) In itu optical LCSM images of Zn plating behavior on Cu foils at 10 mA cm^–2^, (c) Tafel plots of a bare Zn foil before plating and after plating 3 h, and (d) HER plots of a bare Zn foil with a three-electrode system in (a, c, d) ZnSO_4_ electrolytes and (b, c, d) 5% of EGME added ZnSO_4_ electrolytes.

In order to explore the relationship between the morphology of the Zn deposits and the electrochemical behavior, in situ optical LCSM was utilized to monitor the structural evolution of Cu foils during the Zn plating process. Snapshots of a cross section of Cu foil at different stages of deposition, with/without EGMG, are shown in Figure 4a and b. At OCP, the surface of Cu foil is very smooth in both cases, but after a constant current of 10 mA cm^–2^ was applied, uneven zinc crystals appeared on the Cu surface in the pure electrolyte, accompanied by a large number of H_2_ bubbles. Subsequently, after 60 mins of electrodeposition, zinc ions can be seen to deposit preferentially around the as-formed zinc crystal nuclei to form large moss-like protuberances with thicknesses up to ∼15 μm. On the contrary, in the electrolyte with EGME, zinc ions deposited evenly on the Cu foil without any site specific accumulation, eventually forming a homogeneous layer. No H_2_ bubbling or zinc dendritic protrusions could be observed on the surface even after long-term electrodeposition. Hindering HER is extremely beneficial for AZBs, as this is a significant mode of cell degradation and electrolyte loss, making commercialization easier as cells will not have to be designed to counter the effects of gassing. When the above deposited Cu foils were observed under LCSM and SEM (Figure S23), areas where Zn has not deposited could easily be observed for the 0% sample, indicating uneven zinc deposition. For the 5% sample, no unreacted regions could be seen across millimeter scale regions. EDS tests (Figure S24, top view, and Figure S25, cross section) further affirm the uniform deposition.

The positive effect of EGME on the corrosion resistance of Zn foils was verified by Tafel and HER onset potential tests in Figure 4c and d (a detailed analysis can be found in the Supporting Information). These results imply that EGME molecules tend to adsorb on the zinc surface and form a protective layer, much like the solid–electrolyte interface in LIBs. During electroplating, this layer forms via strong chemisorption to inhibit the spontaneous corrosion and undesirable side-reactions.

## Electrochemical Performance

**Figure 5:**
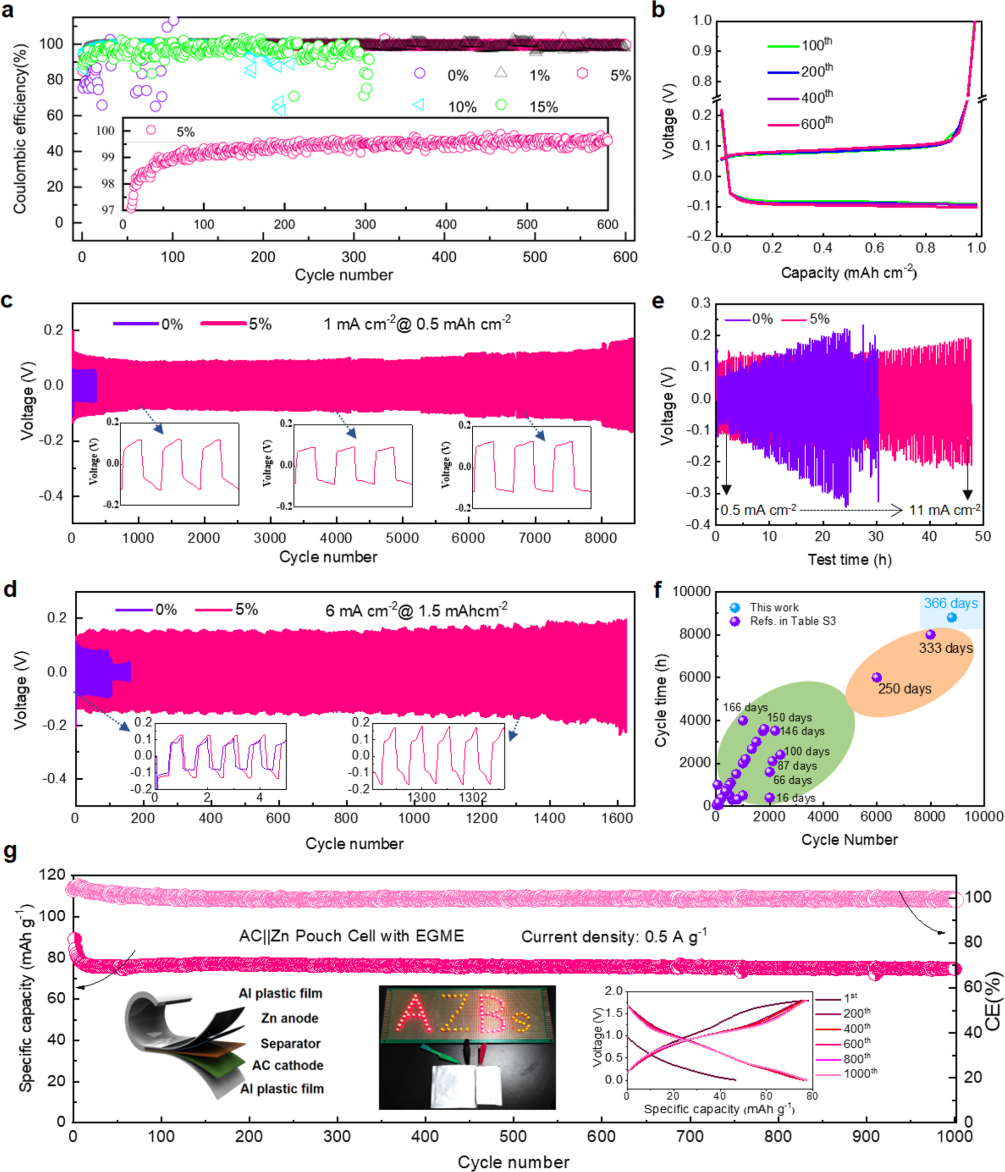
Electrochemical performance of half and full cells. (a) Coulombic efficiency of Cu||Zn cells in electrolytes with different percentages of EGME, from 0% to 15% at a current density of 2 mA cm^–2^, 1 mAh cm^–2^. (b) Polarization curves of Cu||Zn cells in electrolytes with 5% EGME additive at the 100th, 200th, 400th, and 600th cycles, respectively. Long-term cycling performance at (c) 1 mA cm^–2^, 0.5 mAh cm^–2^ and at (d) 6 mA cm^–2^, 1.5 mAh cm^–2^ for Zn||Zn symmetric cells without/with 5% of EGME. Insets are the corresponding high-resolution voltage profiles. (e) Rate performance from 0.5 to 11 mA cm^–2^ for Zn||Zn symmetric cells without/with 5% of EGME. (f) Comparison of the cyclability of Zn||Zn symmetric cells previously reported with various additives and those from this work. (g) Long-term cycling performance for AC||Zn full pouch cell with 5% of EGME additive at a current density of 0.5 A g^–1^. Insets are a digital picture of two pouch cells connected in series lighting a LED board, a schematic diagram of pouch cell and the corresponding galvanostatic charge/discharge curves.

We evaluated the effect of zinc grain refinement by EGME molecules on battery performance through reversible Zn plating/stripping measurements. First, the zinc CE was measured using Zn||Cu coin cells, as shown in Figure 5a, Figure S26, and Figure S27. The CE of Zn||Cu cells with the traditional electrolyte fluctuated greatly, gradually decreasing to below 60% after ∼50 cycles at 2 mA cm^–2^, 1 mAh cm^–2^, likely due to zinc protrusion, the growth of byproducts, or the HER. In contrast, the zinc CE gradually increased and became more stable with the regulation of EGME. With 5% EGME first cycle Zn plating/stripping efficiency was 85.2%, exceeded 97.5% after 10 cycles, and reached an average CE of 99.5% from the 50th to 600th cycle. The corresponding polarization curves are also highly consistent from the 50th to 600th cycles (Figure 5b), further demonstrating the significantly stability. Zn||Cu coin cells with 5% EGME electrolytes also achieved excellent zinc CE at 1 mA cm^–2^, 1 mAh cm^–2^, with an average CE of 99.5% for 400 cycles (Figure S26a), and at 6 mA cm^–2^, 0.5 mAh cm^–2^, with an average CE of 98.5% over 1100 cycles (Figure S26b).

The cycling stability of metallic Zn electrodes was also investigated in Zn||Zn symmetric cells (Figure 5c and d), showing the 5% EGME additive cells could achieve a cycle life of over 8800 cycles (366 days) at 1 mA cm^–2^, 0.5 mAh cm^–2^, compared to ∼200 cycles with the traditional ZnSO_4_ electrolyte, an extremely significant improvement. Even at higher current densities, the Zn symmetric cells exhibited excellent performance: over 1600 cycles at 6 mA cm^–2^, 1.5 mAh cm^–2^ (Figure 5d), and over 200 cycles at 12 mA cm^–2^, 3 mAh cm^–2^ (Figure S28). The rate performance of the symmetric coin cells with the additive was also greatly improved (Figure 5e; detailed analysis can be found in Figures S29 and S30 along with a relevant discussion). The excellent cycling performance of these Zn||Zn symmetric cells in this study compares very favorably with previous reports which report the use of electrolyte additives, exceeding previously reported cycle life and rate performance (Figure 5f and Table S3).

Finally, we employed AC or ZnVO (Zn_0.25_V_2_O_5_) cathodes and a Zn metal anode to assemble commercially viable full cells to evaluate the impact of electrolytes with EGME as an additive (detailed electrochemical information can be found in Figures S31–S34). When tested in cells with a traditional faradaic AZB cathode, namely ZnVO||Zn coin cells (Figure S35a), the EGME additive enables significant cell stabilization. AC cathodes then were studied to avoid complications due to cathode-specific degradation. The full coin cells with EGME offer an ultralong cycle lifespan of over 10000 cycles, with near 100% CE. Importantly, this significant performance enhancement is sustained in practical AC||Zn pouch cells (Figure 5g), where EGME promoted extremely high stability (sustaining near 100% CE after 1000 cycles). Hence, this data suggests that EGME has anode stabilization properties that can be applied widely across ZIB chemistries.

In summary, we report a simple and easily scalable method to fundamentally solve the problem of Zn anode instability via an organic molecule, EGME, which is shown to moderately decrease the surface tension for aqueous electrolytes to form ultrafine nanograins, significantly enhancing cycling reversibility and inhibiting dendrites and hydrogen evolution. Combined in situ/operando experiments and theoretical calculations reveal that EGME both complexes with Zn ions in solution and forms an anode passivation layer, hindering cell degradation. Importantly, through the use of this scalable low percentage additive, a compact, uniform and highly reversible surface is maintained at Zn anodes for long (∼10 000 cycle) lifetimes in full cells at high CE. This methodology and mechanistic understanding therefore offers a promising and simple to implement route for the commercial application of highly reversible, highly safe, and high energy density aqueous batteries at scale and, more widely, offers an approach by which the application of other multivalent battery chemistries could be realized.

## Supporting Information Available

Details about materials synthesis and characterizations, battery assembly and electrochemical measurement, and discussion sections (PDF)
