# Supplementary material for: Nanoscale Ultrafine Zinc Metal Anodes for High Stability Aqueous Zinc Ion Batteries
Source: Nano Lett. 2023 Jan 3;23(2):541–9. doi: 10.1021/acs.nanolett.2c03919 (PMC9881152; doi:10.1021/acs.nanolett.2c03919)
Supplement: Supplementary file 1 — nl2c03919_si_001.pdf [file nl2c03919_si_001.pdf]

Supplementary Information

Nanoscale Ultrafine Zn Metal Anodes for High Stability Aqueous Zinc Ion Batteries

Mingqiang Liu#, Lu Yao#, Yuchen Ji#, Mingzheng Zhang, Yihang Gan, Yulu Cai, Hongyang Li, Wenguang Zhao, Yan Zhao, Zexin Zou, Runzhi Qin, Yuetao Wang, Lele Liu, Hao Liu, Kai Yang, Thomas S. Miller*, Feng Pan*, Jinlong Yang*

[a] Guangdong Research Center for Interfacial Engineering of Functional Materials, College of Materials Science and Engineering, Shenzhen University, Shenzhen 518060 (P. R. China)
* E-mail: yangjl18@szu.edu.cn; https://orcid.org/0000-0001-6065-7272
[b] School of Advanced Materials, Peking University Shenzhen Graduate School, Shenzhen 518055 (P. R. China)
* E-mail: panfeng@pkusz.edu.cn
[c] Electrochemical Innovation Lab, Department of Chemical Engineering, University College London, London, WC1E 7JE, UK
* E-mail: t.miller@ucl.ac.uk
[d] Department of Mechanical Engineering, Imperial College London, London, SW7 2AZ, UK
[e] School of Chemical Engineering and Advanced Materials, The University of Adelaide, North Terrace, South Australia, 5005
[f] Department of Electrical and Electronic Engineering, University of Surrey, Guildford, Surrey, GU2 7XH, UK

[1] M.Q.L., L.Y. and Y.C.J. contributed equally to this work.

Methods

Preparation of electrolyte solutions.

$ZnSO_4 \cdot 7H_2O$ (A.R.) and EGME were provided by Aladdin. To prepare the 3 M $ZnSO_4$ electrolyte $ZnSO_4 \cdot 7H_2O$ was dissolved in deionized water, whereas hybrid electrolyte was obtained by adding EGME solvent with different volume proportions into $ZnSO_4$ electrolyte and stirring.

Synthesis of AC (YP800) and ZnVO electrode.

AC (YP800) was purchased from Guangdong Canrd New Energy Technology Co.,Ltd and mixed with acetylene black and polyvinylidene fluoride (PVDF) at a weight ratio of 7:2:1 with N-methyl-2-pyrrolidone (NMP) as the solvent. The mixture was stirred magnetically overnight to form a uniform slurry. Graphite paper was used as current collector. The as-prepared AC (YP800) slurry was uniformly cast on graphite paper by a doctor-blade, and then dried at 80 °C overnight under vacuum oven to obtain the AC electrode. The mass loading of AC was close to 2 mg cm^{-2}.

$Zn_{0.25}V_2O_5$ (ZnVO) materials was synthesized via a previously reported method,[1] where 1.3 mmol of zinc acetate was dissolved in a water/acetone (volume ratio = 15:1) solution, then added 2 mmol V_2O_5 was added under magnetic stirring. The mixed solution was transferred to a sealed Teflon vessel and kept in an oven at 180 °C for 90 min. The product was collected and washed 3 times with deionized water, and dried overnight at 80 °C. Subsequently, mixing ZnVO, acetylene black, PVDF at a ratio of 7:2:1. The slurry was cast on graphite paper, and dried at 80 °C overnight to obtain the ZnVO electrode.

Cell assembly.

Coin cell (CR2032).

A coin cell (CR2032) is assembled with Zn foil (thickness 52 μm, Φ = 15 mm), the cathode (thickness 103 μm, Φ = 15 mm), fibre paper (55% cellulose + 45% polyester) (thickness 300 μm, Φ = 16 mm) and 100 μL electrolytes.

pouch cell

Zn foil (thickness 52 μm), the cathode (thickness 103 μm) are cut into 3 cm × 3 cm, whereas the paper separator (thickness 300 μm) is cut into 3.2 cm × 3.2 cm and the Al plastic film is around 4 cm × 4 cm. The corresponding mass of each components, including Zn foil, the AC electrode, separator and Al plastic film are 321.0 mg, 58.1 mg, 114.6 mg and 296.2 mg, respectively. The AC||Zn full pouch cells are formed at ambient temperature (25 ℃).

Electrochemical tests.

A Cu||Zn half-cell was used to test the Zn CE by plating Zn on Cu foil under a given current density and deposition time, and then stripping Zn from the Cu foil to a fixed voltage of 1 V. The CE was calculated by the ratio of Zn striping to Zn plating capacity. The in-situ optical measurement was performed under an optical microscope with a separator-free Cu||Zn half-cell.

For the measurement of the HER, a three-electrode system was used with Ti foil as the working electrode and counter electrodes and an Ag/AgCl reference electrode. The test was carried out using a CHI604E electrochemical workstation (Chenhua, Shanghai) with a scan rate of 0.1 mV s^{-1}. The Tafel plot was collected on the CHI604E electrochemical workstation under a scan rate of 0.4 mV s^{-1} in a three-electrode electrolysis bath with Zn foil as the working electrode, a Pt sheet as counter electrode and an Ag/AgCl reference electrode. The Zn^{2+} stripping/plating behaviors with/without EGME was investigated on Ti||Zn cells using CV measurements at a scan rate of 0.5 mV s^{-1}. And the CV curves of ZnVO was tested between 0.1 V and 1.6 V on ZnVO||Zn batteries. A Zn||Zn symmetric cell was used to measure the stability of the Zn anode at a given current density and capacity. For the AC||Zn full-cells, the AC electrode (see above) and Zn foil were separated by paper separator (55% cellulose + 45% polyester) in both the coin- and pouch-cell format. A given current density and voltage of 0.1 to 1.8 V was applied for cell charge and discharge. In addition, all electrochemical tests are carried out at ambient temperature.

Materials characterization.

Transmission electron microscopy (TEM, Titan G260–300, FEI) was applied to characterize the sample morphology and to collect selected area electron diffraction (SAED) patterns. Scanning electron microscopy (SEM, SUPRA 55 SAPPHIRE, Carl Zeiss AG, Germany) and energy dispersive spectrometry (EDS,51-XMX1004, Oxford Instruments Inc., America) were used to observe the surface morphology and elemental distribution of the Zn foil after stripping and Cu foil after plating. Powder X-ray diffraction (XRD, D8 ADVANCE, Bruker Co., Germany) was applied to analyze Zn deposits using a Cu Kα source, λ=1.541 Å, between 2θ from 3° to 60°. Thermogravimetry measurements (TGA, SII STA7300 analyzer) were performed under a nitrogen atmosphere to illustrate the purity and decomposition temperature of EGME. ^{1}H NMR spectra was recorded on a Bruker AVANCE III 400 MHz NMR spectrometer with D_2O as the solvent. X-ray photoelectron spectroscopy (XPS, ESCALAB 250) was used to explore the electronic structure and composition of zinc deposits.

Discussion of NMR measurements.

The real interaction between EGME molecules and zinc ions in typical aqueous solutions was measured by the nuclear magnetic resonance (NMR). The ^{1}H NMR and TGA characterization of EGME molecules are shown in Figure S3 and Figure S4,

respectively, the three peaks at 3.59, 3.44 and 3.26 are ascribed to EGME molecule. Besides, ^{1}H NMR spectroscopy of 3 M $ZnSO_4$ with different percentage of EGME solvent from 0% to 15% in D_2O confirmed that EGME molecules have a function of weakening the solvation interaction between Zn^{2+} and H_2O molecules (Figure 1a). The ^{1}H peak of pure D_2O lies in 4.6773 ppm. After dissolving 3 M $ZnSO_4$ into D_2O, the ^{1}H peak shift to 4.7001 ppm, due to a reduced electronic density and proton shielding around water molecules, which consistent with typical solvation sheath between Zn^{2+} and D_2O molecules[2]. Afterwards, with the augment of EGME solvents, the ^{1}H peak gradually move forward from 4.6982 ppm (5%) to 4.6970 ppm (10%) and 4.6954 ppm (15%), respectively, denoting more water molecules are freed from the solvation shell of $[Zn(H_2O)_6]^{2+}$, which is compatible well with the coordination model between EGME molecules and Zn^{2+} from calculation results. The results show that water molecules from $[Zn(H_2O)_6]^{2+}$ solvation sheath are replaced by the organic molecules, and the electrolysis amount of active water on the electrode surface is dramatically decreased.

Discussion of the charge density.

The charge density (Figure 1e) showed that electrons tend to transfer from EGME to Zn and hence strongly chemisorb on the interface, suggesting EGME can promote the development of a passivation layer to minimize corrosion from the direct contact of Zn metal with free water.

Discussion of XRD patterns.

To further explore the interfacial composition of the Zn deposits, metal anodes were examined using X-ray diffraction (XRD, Figure 3g). While all expected Zn metal peaks were present for both electrodes, the one cycled in pure $ZnSO_4$ also exhibited a number of additional peaks, with three major contributions at $2\theta = 8.07°$, 12.23° and 13.96° that can be assigned to the by-product $Zn_4SO_4(OH)_6{\cdot}5H_2O$ (PDF#39-0688), consistent with the EDS and TEM results. While very small peaks can be resolved in the same positions for the electrode cycled with the EGME additive, demonstrating minimal amounts of by-products are produced, showing that EGME has a significant ability to minimize parasitic reactions during cycling.

Perhaps more intriguingly, the XRD data (Figure S20) also shows that the electrolyte additive has an impact on the intensity ratio of the (002):(101) and (100):(101) crystallographic planes of the zinc. A schematic diagram of the (101), (002) and (100) planes are shown in Figure S22. The (002) constitutes 27.8% of the (101) intensity (without EGME), changing to 52% (with EGME), and the (100) changes from 21.6% (in the $ZnSO_4$ electrolyte) to 32.2% (in the $ZnSO_4$-EGME electrolyte), indicating that the addition of EGME has influenced the preferred orientation of zinc crystal growth from (101) to (002) and (100) lattice planes via interfacial chemisorption forming a barrier layer. This is consistent with the greatly reduced adsorption energy calculated in Figure 1d and Figure S6.

Discussion of typical nucleation equations.

Furthermore, according to the Sand's time model[3, 4] (Equation 4), the growth of Zn dendrites can be effectively refined by directly manipulating the electrolyte to prolong the Sand's time[5] by increasing the initial electrolyte concentration, decreasing the effective current density or tuning the mobilities of cations and anions. Thus, high concentration 3 M $ZnSO_4$ was used as initial electrolyte in this study.

$$\tau = \pi D \frac{eC_0(u_a + u_c)^2}{2Ju_a} \tag{4}$$

where, τ stands for the dendrite formation time and D, e, C_0, j, μ_a and μ_c represent the diffusion constant, the electronic charge, the initial concentration of the electrolyte, the effective current density, the anionic and cationic mobility, respectively.

In addition, classical equations for homogeneous nucleation can be used to describe the relationship between the size of electrodeposited nuclei and NOP (Equation 5 and

6, Figure 1g).[6] [7] Here, r is the radius of zinc nuclei, ΔG_v is the free energy change per volume, γ is the surface energy of the interface, F is Faraday's constant and V_m is the molar volume of zinc.

$$\Delta G_{nucleation} = -4/3\pi r^3 + 4\pi r^2\gamma \quad (5)$$

$$\Delta G_v = F|\eta|V_m \quad (6)$$

The Gibbs free energy ($\Delta G_{nucleation}$) for forming a spherical nucleus is the sum of its volume and surface free energies (Equation 5), whereas the nuclei size is proportional to the inverse of overpotential and the number density of nuclei is proportional to the cubic power of overpotential[8] (Figure 1g).

Discussion of SEM images of zinc electrodeposits (Figure 2c and Figure 2d).

SEM images further show the differing microstructure of zinc electrodeposits between 10 and 600 mins. As shown in Figure 2c, after galvanizing on Zn foil in the traditional electrolyte, irregularly shaped small zinc crystals can be clearly seen, including hexagonal, polygonal and round flakes. As time progressed, Zn deposits preferentially grew along the initial nuclei and the hexagonal Zn flakes became bigger and more randomly arranged on the Cu surface. Eventually a large number of zinc flakes became stacked on top of each other and formed thick plates, finally growing into larger and densified dendrites, consistent with the AFM results. TEM images of a single particle in Figure S15a and b confirmed the density of the micron-scale hexagonal zinc sheets. The average size of Zn flakes after 300 minutes of deposition was 7.0 um (Figure S16a, b and c), a size that would certainly cause damage within an operational AZBs. However, by regulating deposition using EGME (Figure 2d), the initial zinc electrodeposits were in the form of small spheroids, which grew evenly over the first 60 minutes. When the electrodeposition time increased to 300 minutes, the zinc crystals grew into prolate spheroid (i.e. rice-shaped) nano-pellets of ~250 nm (Figure S16d, e and f), and then self-assembled into bigger nanocrystals of ~ 400 nm after 600 minutes (Figure 2(d4)). TEM images clearly demonstrated that the rice-like crystals are loose and porous, and are likely assembled from aggregated spherical crystal nuclei (Figure S15c and d). The evolution of zinc deposition morphology over time is summarized in Figure S17.

Discussion of Tafel and HER plots.

The positive effect of EGME on the corrosion resistance of Zn foils was verified by Tafel and HER onset potential tests (Figure 4c and 4d). Compared with the corrosion potential of -0.894 V and current density of 7.362×10^{-4} A cm^{-1} for bare Zn foil in pure electrolytes, values of -0.860 V and 3.006×10^{-5} A cm^{-1} were obtained for a bare Zn foil in the 5% EGME electrolyte, showing that the adsorption of additives plays an important role in reducing corrosion. Surprisingly, the corrosion potential and current density of the Zn foil after plating in the electrolyte with the additive changed further to -0.846 V and 1.236×10^{-5} respectively, possibly indicating a transition from physisorption to chemisorption. The HER onset potential of pure electrolytes, measured in a three-electrode system, was found to be -0.99 V, 43 mV more positive than that measured in the EGME mediated electrolyte (-1.03 V).

Discussion of the impact of excess EGME on Zn metal electrochemistry.

However, the addition of excess EGME (10% and 15%) was found to negatively impact Zn stability to ~300 cycles. The corresponding cycle overpotentials were 53.8 eV (0% EGME), 142.2 eV (1%), 171.1 eV (5%), 196.0 eV (10%) and 245.1 eV (15%), as derived from the data shown in Figure S27. This shows that the optimal level of EGME can increase the overpotential sufficiently to help to form even zinc nuclei and significantly improve Zn stripping/plating, however excessive addition likely leads to excessive interfacial charge-transfer resistances, reducing the effect of the organic molecules on zinc ion electrodeposition. A comparison of the electrochemical

impedance spectra (EIS) (Figure S29) further reflected the rise in impedance and polarization with the gradual addition of EGME.

Discussion of the polarization curves of Zn||Zn symmetric cells.

The cycling stability was extended to 2980 cycles (1% EGME), 3808 cycles (5%), 1700 cycles (10%) and 538 cycles (15%) at 4 mA cm^{-2}, 0.5 mAh cm^{-2}, while the symmetric cells without additive underwent an abrupt voltage drop after 402 cycles, indicating a short-circuit. Insets show the high-resolution voltage profiles during the initial cycles (Figure S28a). With the addition of EGME, a gradual increase in over-potential from 114 mV for 0%, to 136 mV (1%), 143 mV (5%), 148 mV (10%) and 171 mV (15%) was again found, indicating the raise in interfacial charge transfer impendence. Importantly, the polarization overpotential profiles all include sloping curves, indicating that the symmetric cells do not suffer from soft shorts[9].

Discussion of the rate performance of Zn||Zn symmetric cells.

Although the batteries without the additive were found to have a lower overpotential during the initial cycles at low current densities, the polarization curves increased rapidly when the current density increased, eventually short circuiting at 6 mA cm^{-2}. However, the symmetric cells with the EGME were able to cycle to 10.5 mA cm^{-2} (1%), 11 mA cm^{-2} (5%), 9 mA cm^{-2} (10%), 7 mA cm^{-2} (15%) without short circuits occurring, giving further proof that the optimal amount of EGME is ~5%.

Discussion of the long-term cycle performance of ZnVO||Zn and AC||Zn cells.

When tested in cells with a traditional faradaic AZB cathode, namely ZnVO||Zn coin cells (Figure S32 and Figure S35a), the EGME additive enables significant cell stabilization. While the cells with the pure electrolyte and 5% EGME additive offered similar first cycle capacities (369 and 387 mAh g^{-1}, respectively), the pure electrolyte cell displayed rapid capacity loss. The comparison of charge-discharge curves after 200 cycles (Figure S35b) exhibits a large capacity difference. Although the cell with EGME can also be seen to lose capacity over its lifetime, per-cycle capacity decay is significantly reduced (~0.17 % over vs ~0.8 %) and much extended cell lifetimes are offered, suggesting cathode degradation may be a significant contributor here. AC cathodes then were studied to avoid complications due to unrelated cathode degradation. CVs of representative AC||Zn full cells including electrolytes without (Figure S31c) / with (Figure S31d) EGME are shown at different scan rates from 0.5 mV s^{-1} to 10 mV s^{-1}. There is no significant difference between the two, indicating that EGME does not contribute to the mechanism of charge storage, but simply impacts interfacial stability. EIS tests are compared in Figure S33, it can be seen that full cells with the EGME additive have faster ionic conductivity, which may be due to the more stable interface between the electrode and the hybrid electrolyte. Wetting angles, shown in Figure S34a-b, also demonstrate the EGEM electrolyte offers a better interfacial wettability, which is beneficial to the capacity of cathode materials. Together with the proven ability of EGME to hinder dendrites and adverse side reactions, these data offer good evidence for the reason AC||Zn full cells are able to offer both a higher discharge capacity and significantly enhanced rate capability and long-term cycling performance (Figure S34c, Figure S35c and Figure 5g). The galvanostatic charge-discharge curves with EGME at both rates (Figure S34d) and long-term cycles (insets in Figure 5g) demonstrate narrower polarization and better reversibility compared to the gradually increasing polarization and deteriorating cycle stability in pure electrolyte (Figure S35d). Specially, in long-term cycling performance tests the AC||Zn coin cells in pure electrolytes have only 50% capacity retention after ~2000 cycles, whereas the full coin cells with EGME offer an ultra-long cycle lifespan of over 10000 cycles, with near 100% CE. Importantly, this significant performance enhancement is sustained in practical AC||Zn pouch cells (Figure 5g), where electrolytes containing EGME promote extremely high stability (sustaining near 100% CE after 1000 cycles).

Density functional theory calculations.

The geometries of water and EGME coordination with zinc ions was obtained using B3LYP-D3 using Gaussian 16 program package [25 26 27]. We used the SDD basis sets for zinc atoms and 6-311+G** basis sets for hydrogen, carbon and oxygen atoms[28 290 30]. Solvation energies were calculated by a self-consistent reaction field using the SMD model[31]. Molecule orbitals were plotted by Multiwfn software and visual molecular dynamics (VMD)[32]. The diagram of cluster structures was plotted by CYLview.
The calculation of water and EGME adsorption energies on the Zn slab are performed based on density functional theory (DFT) applying the Vienna ab initio simulation package (VASP) with projector augmented wave (PAW) potentials[33 34]. The exchange-correlation part of the density function was utilized within the generalized gradient approximation (GGA) of Perdew-Burke-Ernzerhof (PBE) functional[35]. The Van der Waals interactions were taken into consideration by using Grimme's correction[36]. Energy cutoff values were set to 520 eV for structural optimization. Convergence thresholds were set to 1.0×10-5 eV per atom in energy and 0.02 eV Å-1 in force. We constructed three different lattice plane of Zn slab ((002), (100), (101)) with the bottom layer fixed. Also, a (2 × 2 × 1) k-point grid determined by Monkhorst-Pack method were used to sample the Brillouin zones[37]. All the graphs of atomic structures were plotted using VESTA.

Discussion of Electrostatic potential (ESP).

Electrostatic potential (ESP) (Figure 1c and Figure S5) further showed that replacing H_2O molecules in $[Zn(H_2O)_6]^{2+}$ with EGME (replacing between one and three H_2O) consistently decreased the ESP with a minimum for $[Zn(EGME)_3]^{2+}$, which are mainly the contribution of electron clouds on dioxygen functional groups of EGME molecules. The smaller minimum ESP values demonstrate that Zn^{2+} are favorable to be ionized and rapid migration to the electrode surface for deposition to form zinc metal.[10]

Supplementary Figures

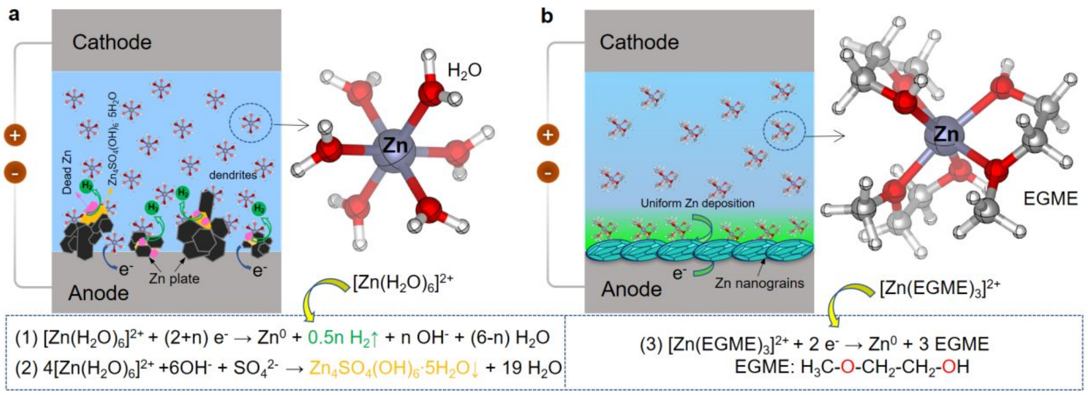

Figure S1. **a**, **b**, Schematic diagrams of the chemical and electrochemical reactions that occur on the Zn metal surface in electrolytes without/with the EGME additive. The corresponding coordination models of $[Zn(H_2O)_6]^{2+}$and $[Zn(EGME)_3]^{2+}$ are presented on the right.

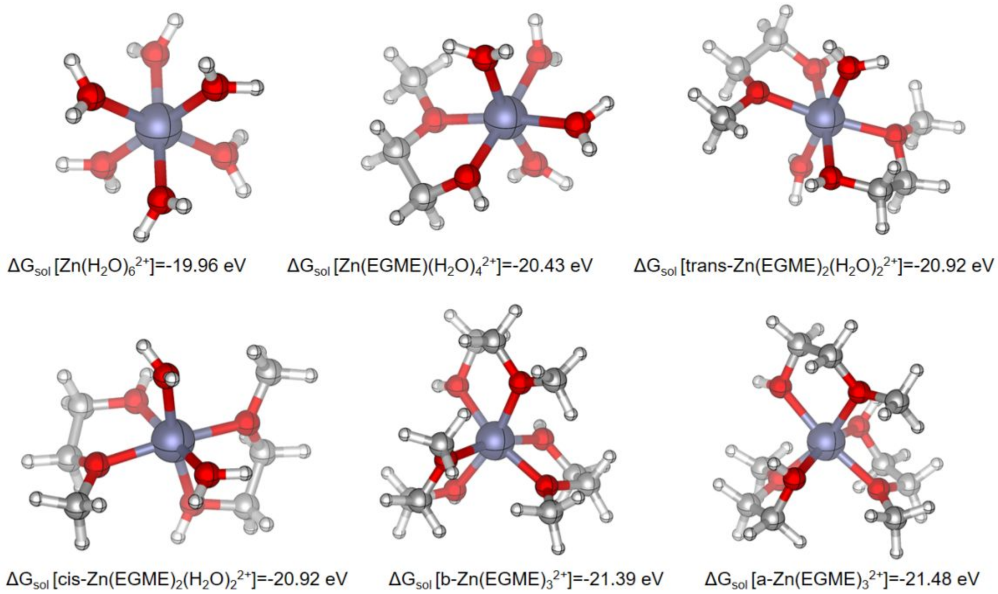

Figure S2. Binding energy of Zn^{2+} coordinated with H_2O and EGME molecules from DFT calculations.

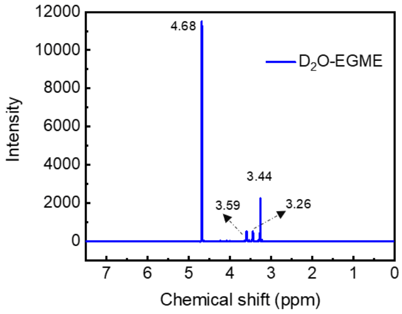

Figure S3. ^{1}H NMR spectroscopy of EGME in D_2O.

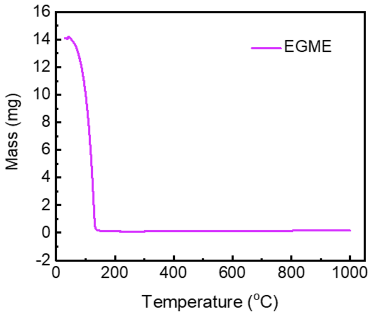

Figure S4. TGA of pure EGME.

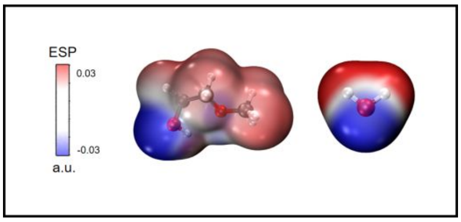

Figure S5. Electrostatic potential mapping of EGME (left) and H_2O (right) molecule structures.

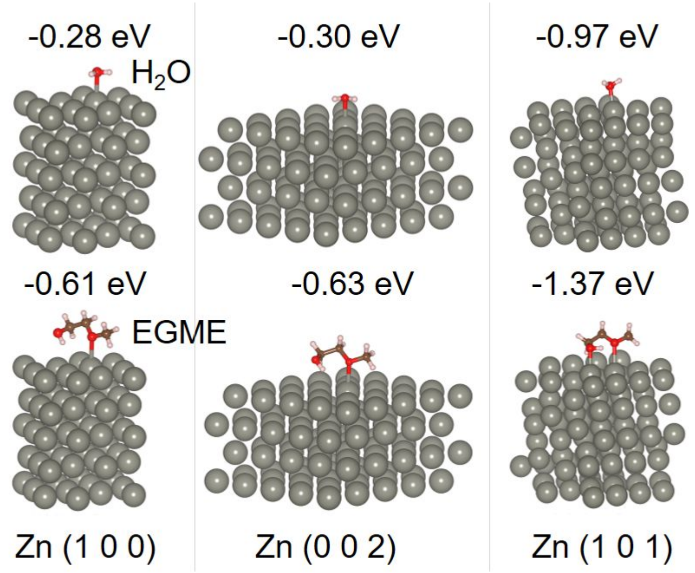

Figure S6. Adsorption energies of H_2O and EGME molecules on different Zn crystal planes, including (002), (100), (101).

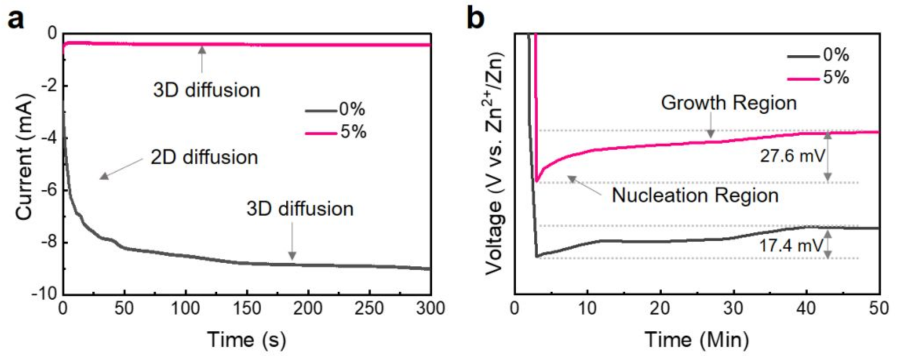

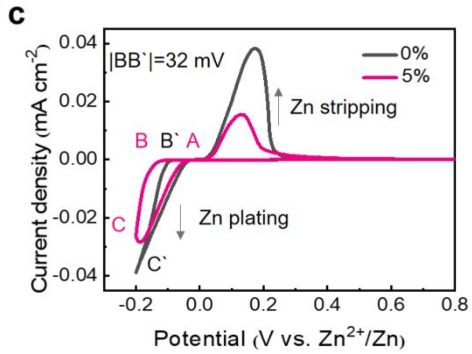

Figure S7. (a) Chronoamperometric curves of Zn electrodeposition at -150 mV overpotential, (b) voltage curves of Zn plating on Cu foil at 0.5 mA cm^{-2} and (c) cyclic voltammetry (CV) plots of zinc plating/stripping in the two electrolytes.

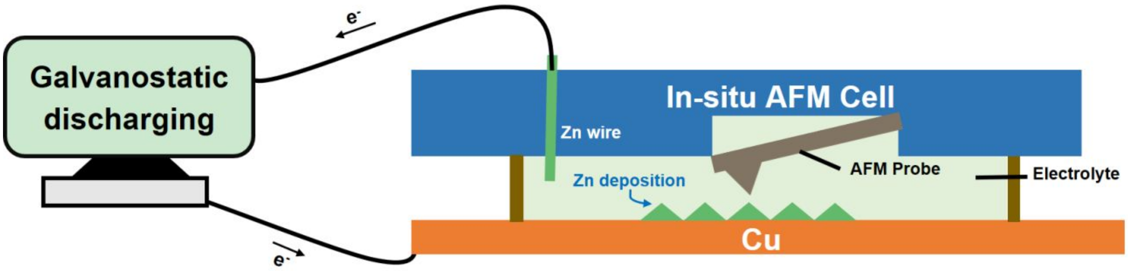

Figure S8. Schematic showing the in-situ AFM cell.

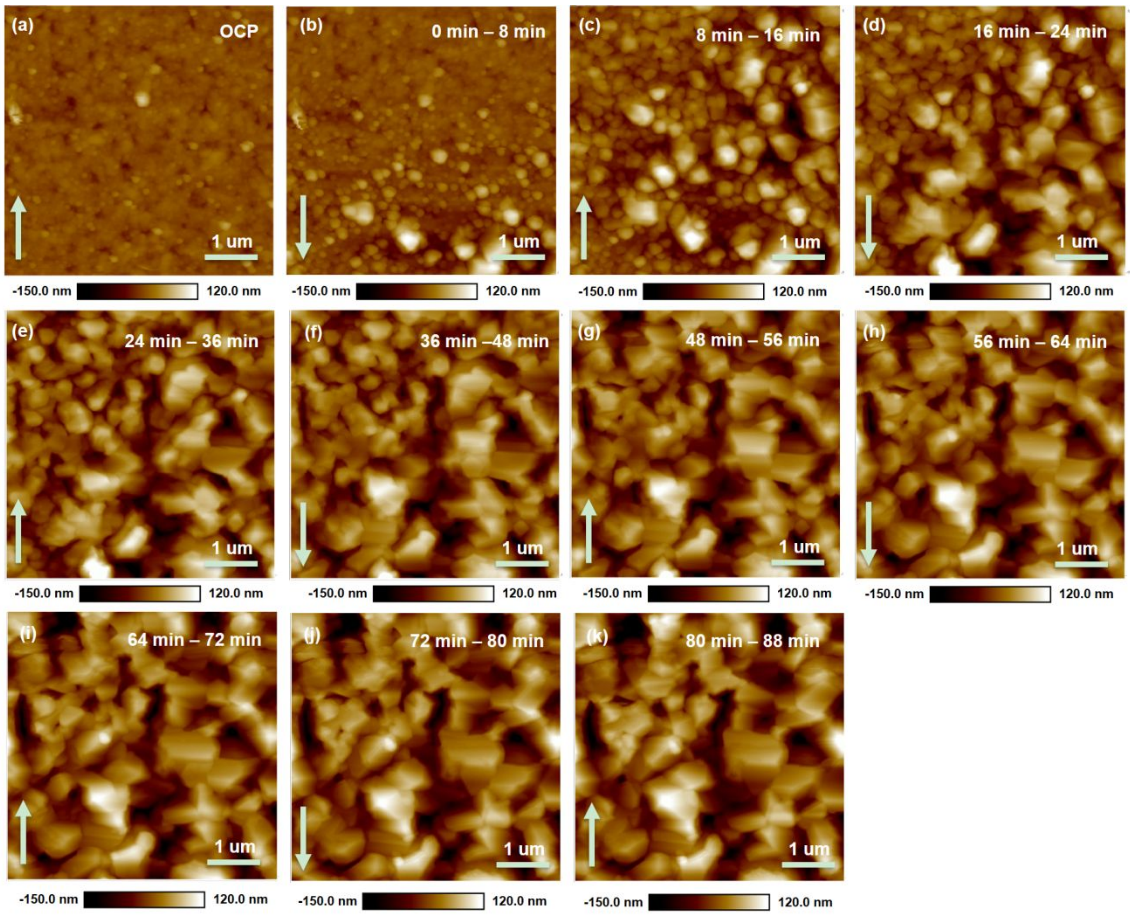

Figure S9. In situ AFM images of zinc nucleation and growth processes in a pure $ZnSO_4$ electrolyte, captured during the galvanostatic electrodeposition process from 0 minutes to 88 minutes at a current density of 0.1 mA cm^{-2}.

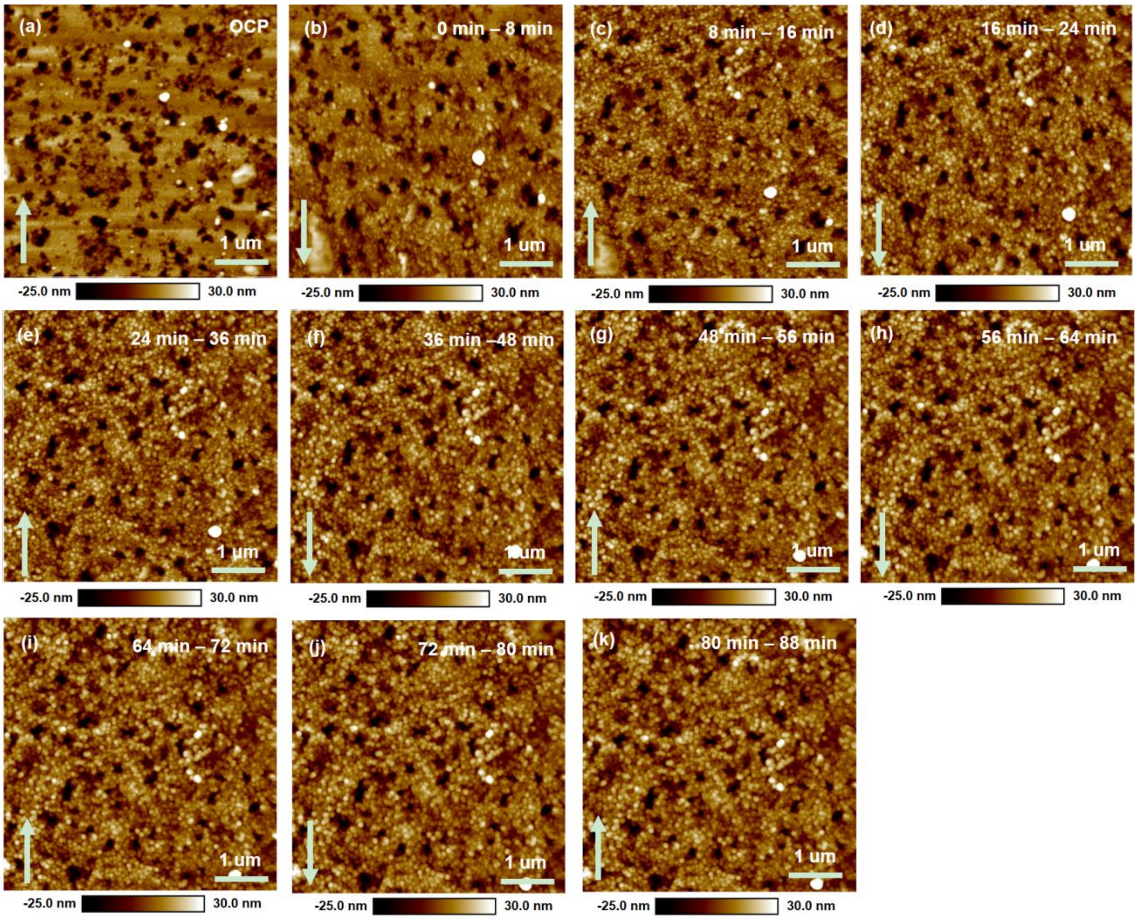

Figure S10. In situ AFM images of zinc nucleation and growth processes in a $ZnSO_4$ electrolyte with an EGME additive, captured during the galvanostatic electrodeposition process from 0 minutes to 88 minutes at the current density of 0.1 mA cm^{-2}.

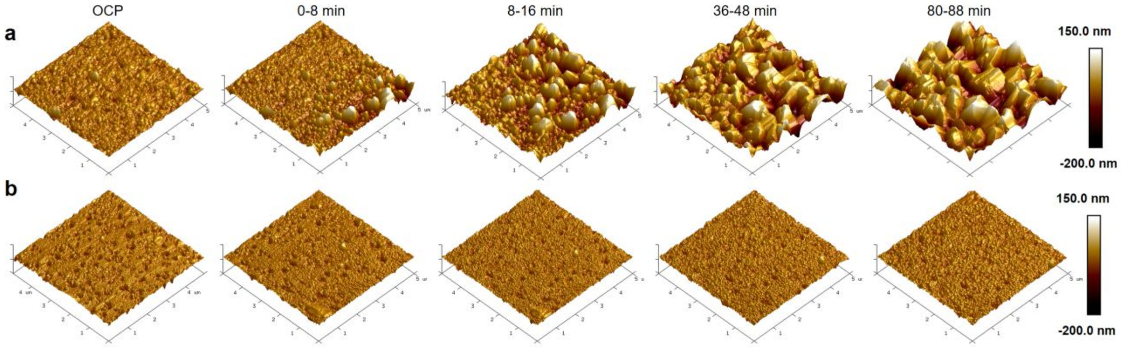

Figure S11. a, **b**, 3D in situ AFM images of zinc nucleation and growth process in electrolytes without/with EGME, selected from Figure S9 and Figure S10.

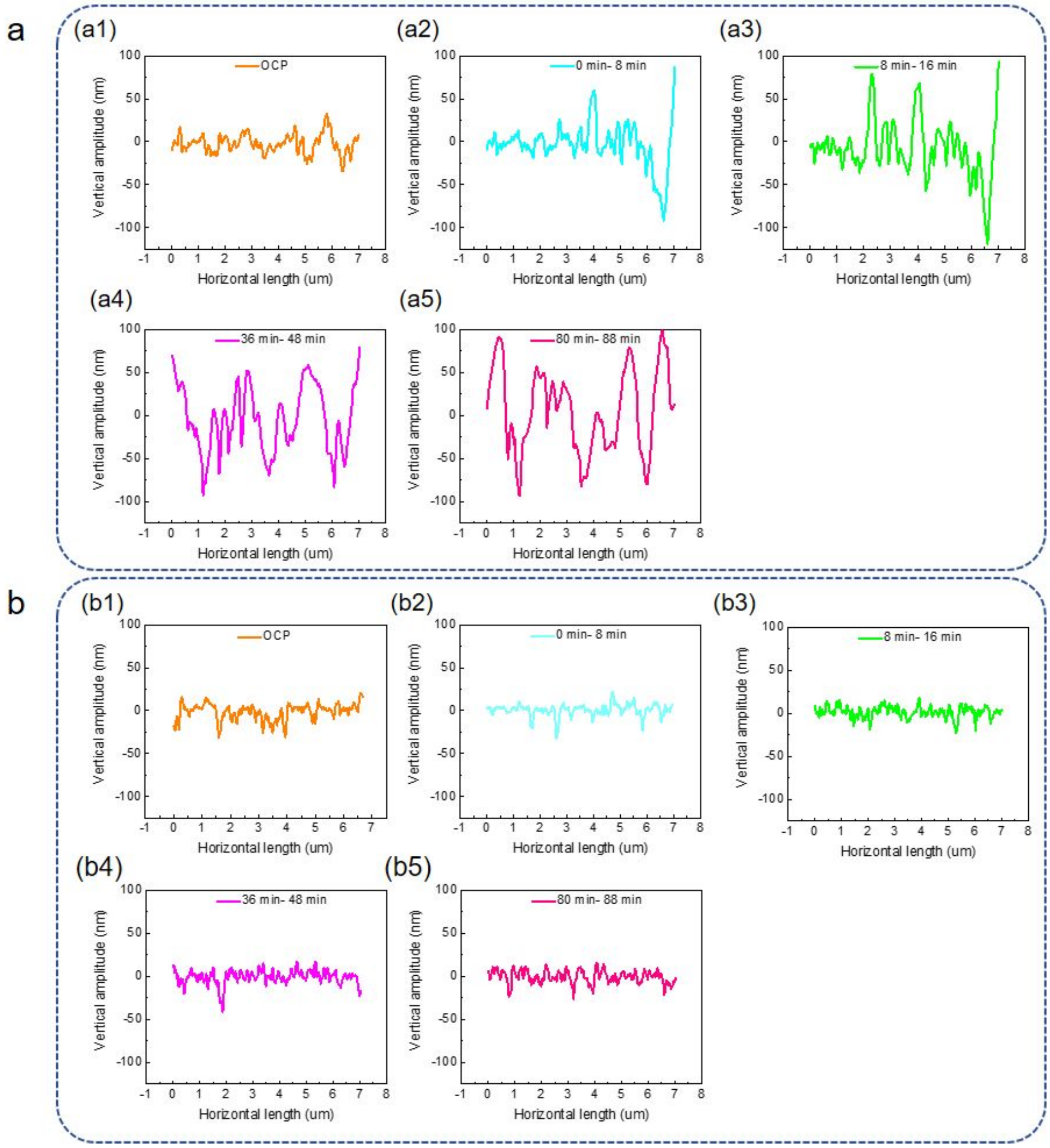

Figure S12. a, **b**, Corresponding line profiles extracted from diagonal cross sections of in situ AFM images of zinc nucleation and growth process in electrolytes without/with EGME from Figure S11.

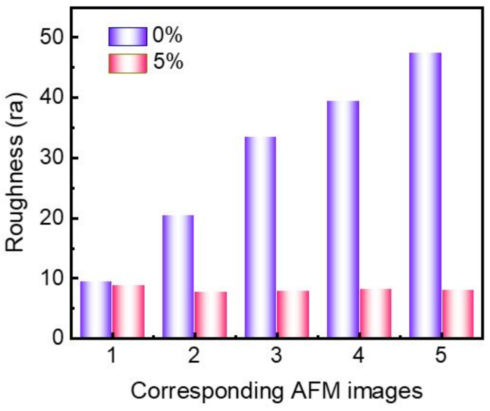

Figure S13. Surface roughness measured from in situ AFM images of zinc nucleation and growth process in electrolytes without/with EGME from Figure S11.

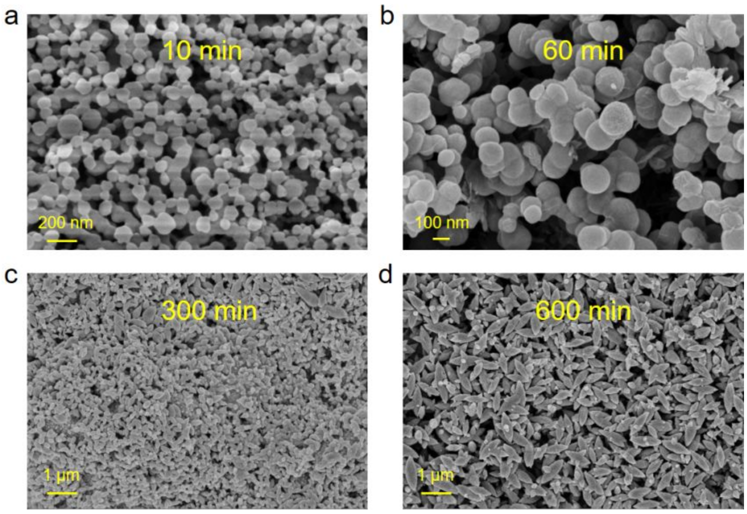

Figure S14. SEM images of Zn electrodeposits at (a) 10, (b) 60, (c) 300 and (d) 600 minutes in electrolytes with 5% of EGME.

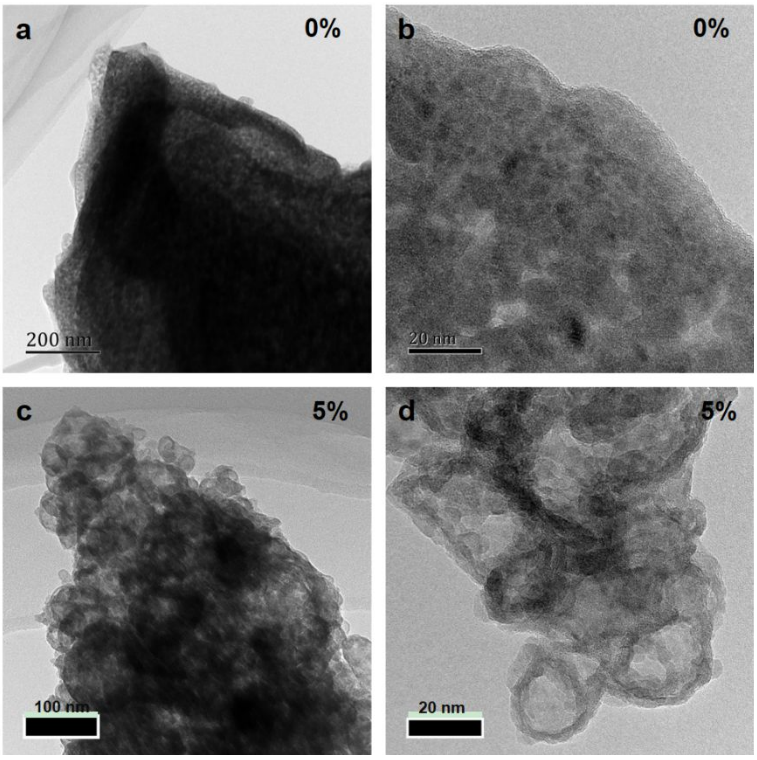

Figure S15. TEM images of single zinc particles after electroplating in $ZnSO_4$ electrolytes without **a**, **b**/with **c**, **d** EGME.

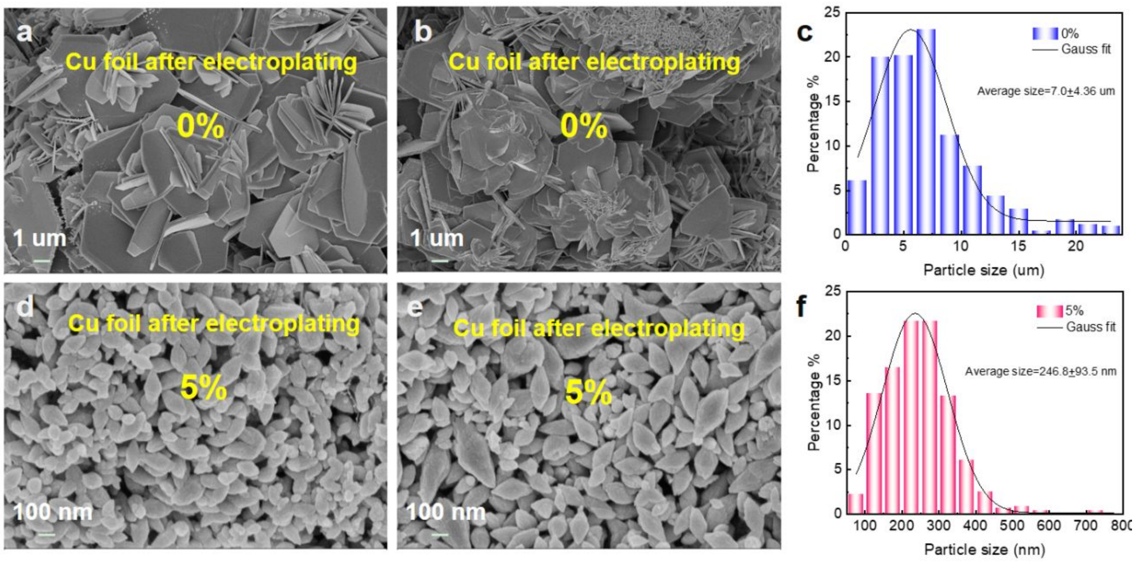

Figure S16. a, b, c, e, SEM images of Zn deposits in $ZnSO_4$ electrolytes without /with EGME. The corresponding particle size distribution of Zn deposits without **d**/with **f** EGME.

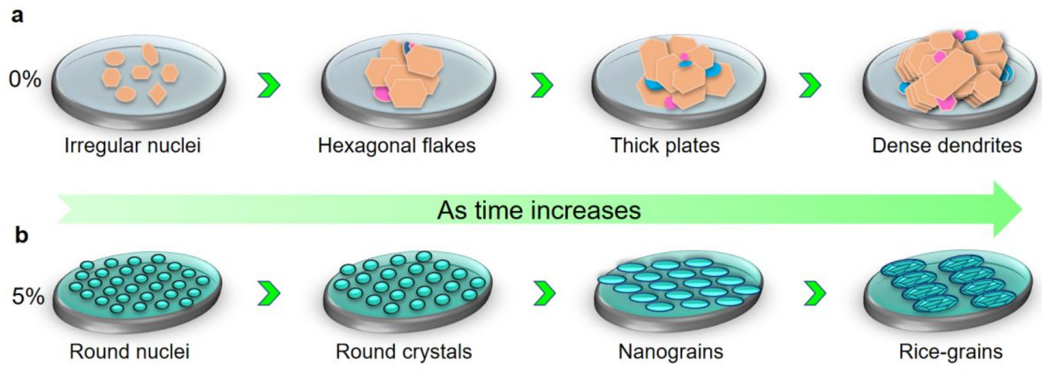

Figure S17. a, b, Schematic diagram of the change of zinc deposition morphology with time in electrolytes without/with EGME.

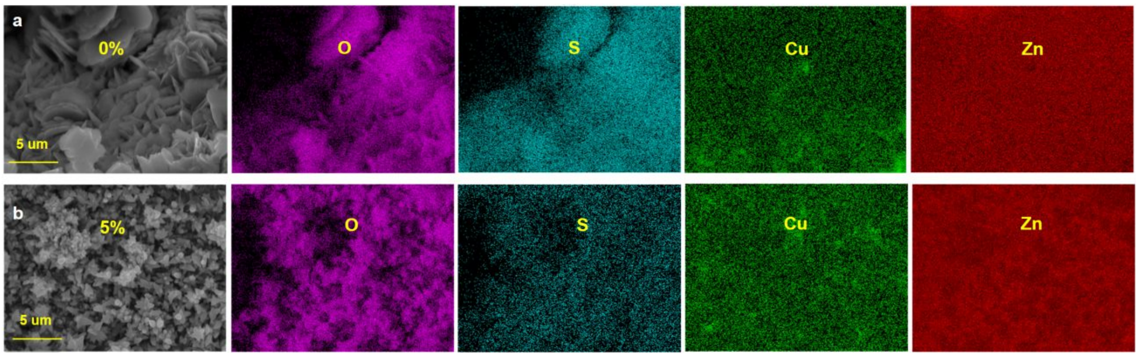

Figure S18. a, b, EDS of Zn deposits on Cu foil after plating in electrolytes without/with EGME.

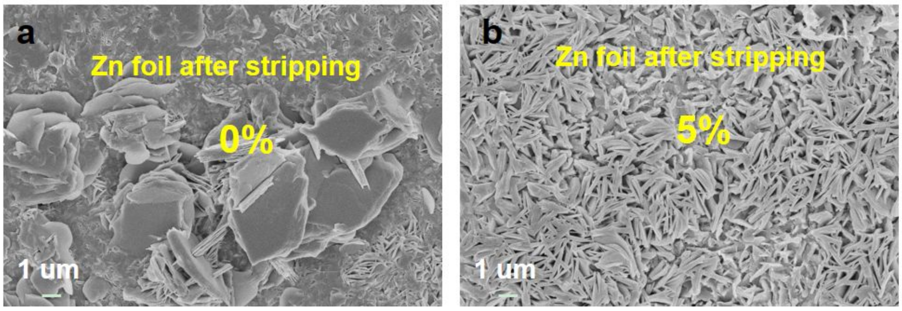

Figure S19. a, b, SEM images of Zn foils after stripping in electrolytes without/with EGME.

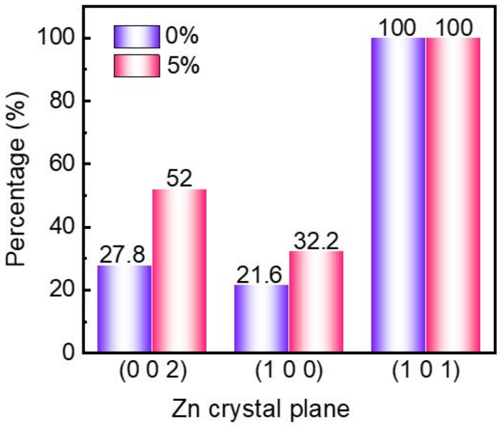

Figure S20. Corresponding intensity comparison of Zn crystal planes include (002), (100) and (101) of XRD patterns.

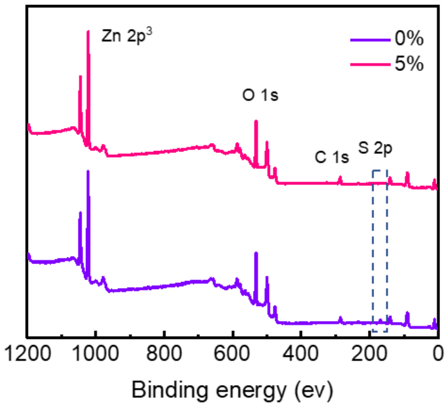

Figure S21. XPS spectra of cycled Zn metal in electrolytes with/without EGME.

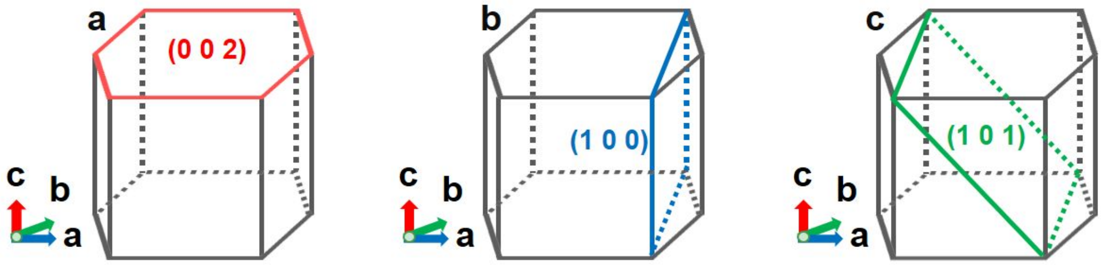

Figure S22. Schematic diagram of the **a** (101), **b** (002) and **c** (100) planes of Zn.

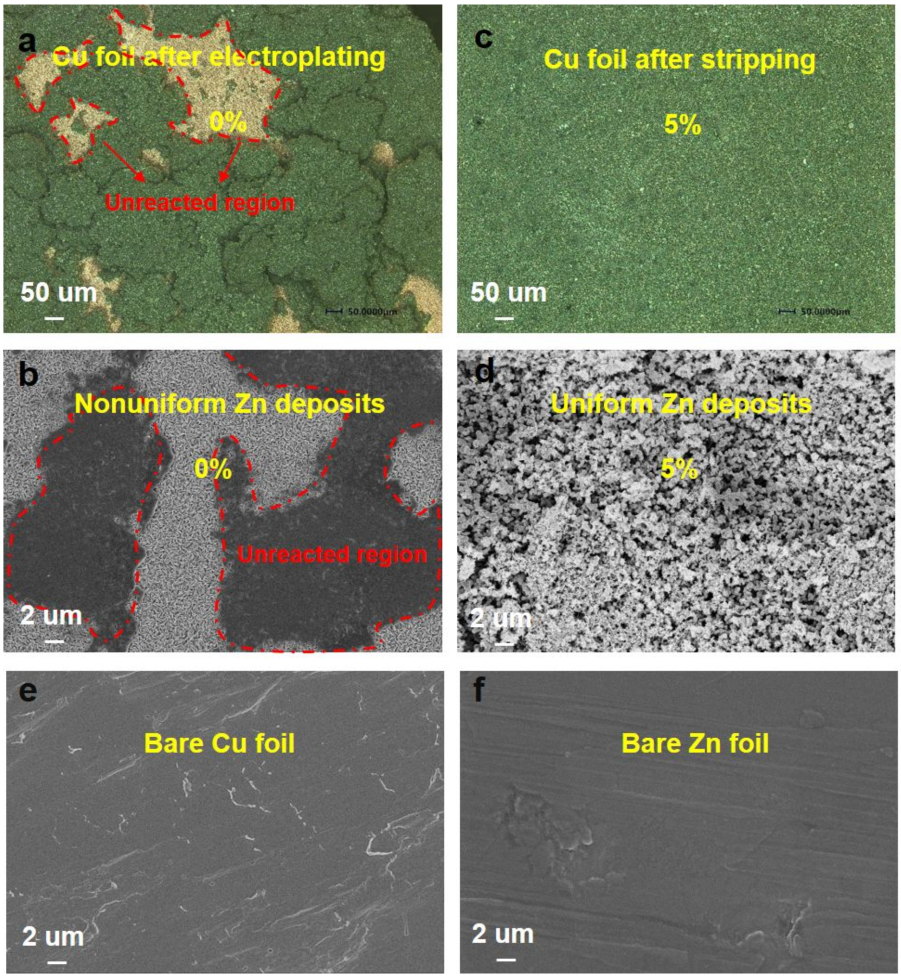

Figure S23. LCSM and SEM images of zinc electroplating on Cu foil in electrolytes without **a**, **b**/with **c**, **d** EGME at the current densities of 10 mA cm^{-2}, 20 mAh cm^{-2}. **e, f,** SEM images of bare Cu foil and bare Zn foil.

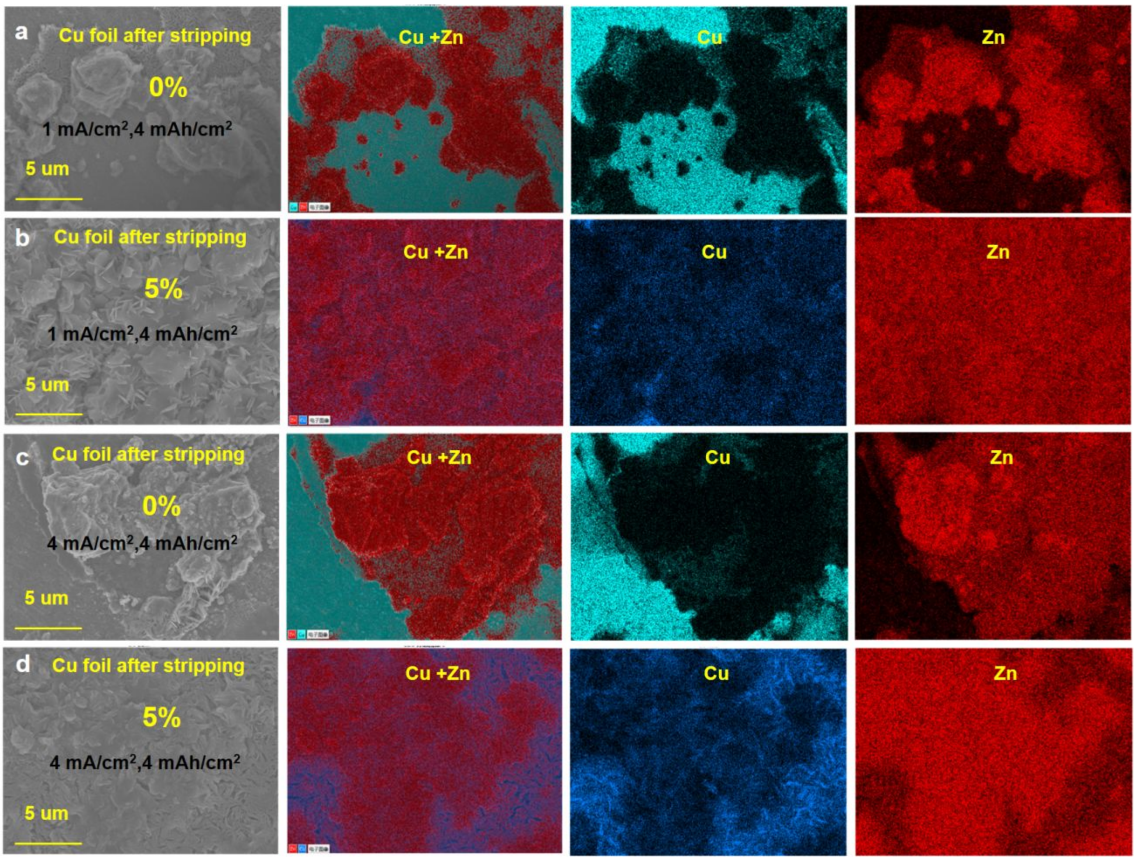

Figure S24. a, b, c, d, EDS of Zn deposits on Cu foil after plating in electrolytes without/with EGME at current densities of 1mA cm^{-2}, 4mAh cm^{-2} and 4mA cm^{-2}, 4mAh cm^{-2}.

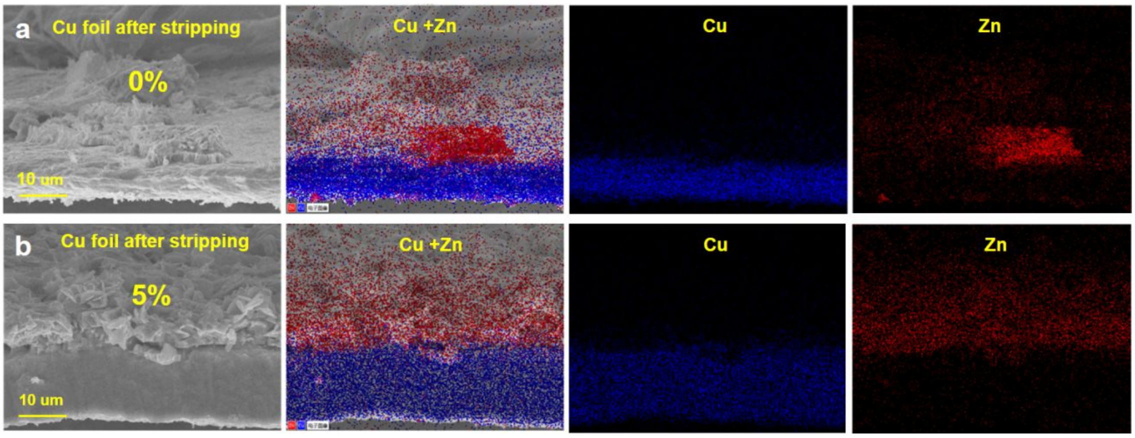

Figure S25. a, b, Cross sections of the Zn deposits on Cu foils after plating in electrolytes without/with EGME at current densities of 1mA cm^{-2} and 4mAh cm^{-2}.

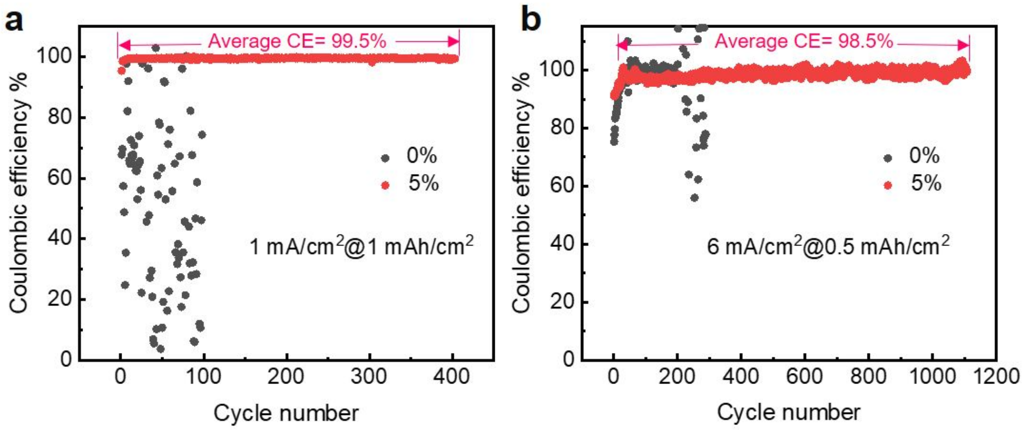

Figure S26. CE of Cu||Zn cells in electrolytes without/with EGME at **a,** 1 mA cm^{-2}, 1 mAh cm^{-2} and **b,** 6 mA cm^{-2}, 0.5 mAh cm^{-2}, respectively.

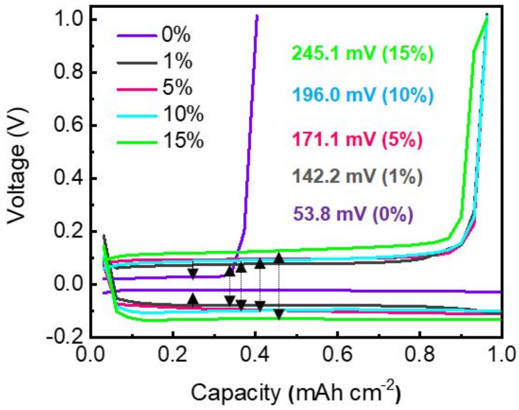

Figure S27. Comparison of Cu||Zn cell overpotentials in electrolytes with different percentages of EGME at 2 mA cm^{-2}, 1 mAh cm^{-2}.

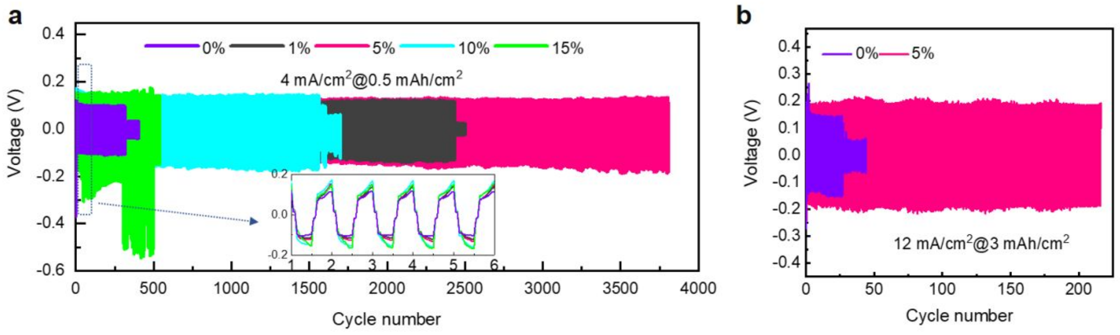

Figure S28. Long term cycling performance of Zn||Zn symmetric cells in electrolytes with different percentages of EGME, from 0% to 15% (insets are corresponding high-resolution voltage profiles) at a current density of (**a**), 4 mA cm^{-2}, 0.5 mAh cm^{-2} and (**b**) 12 mA cm^{-2}, 3 mAh cm^{-2}.

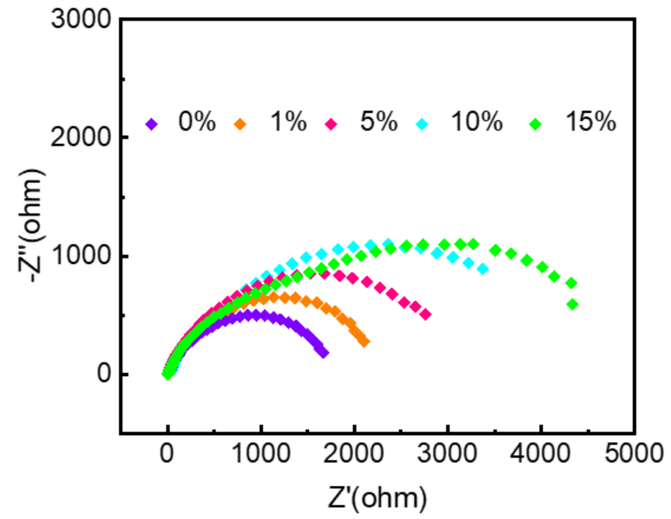

Figure S29. EIS of Zn||Zn symmetric cells in electrolytes with different percentages of EGME, from 0% to 15%.

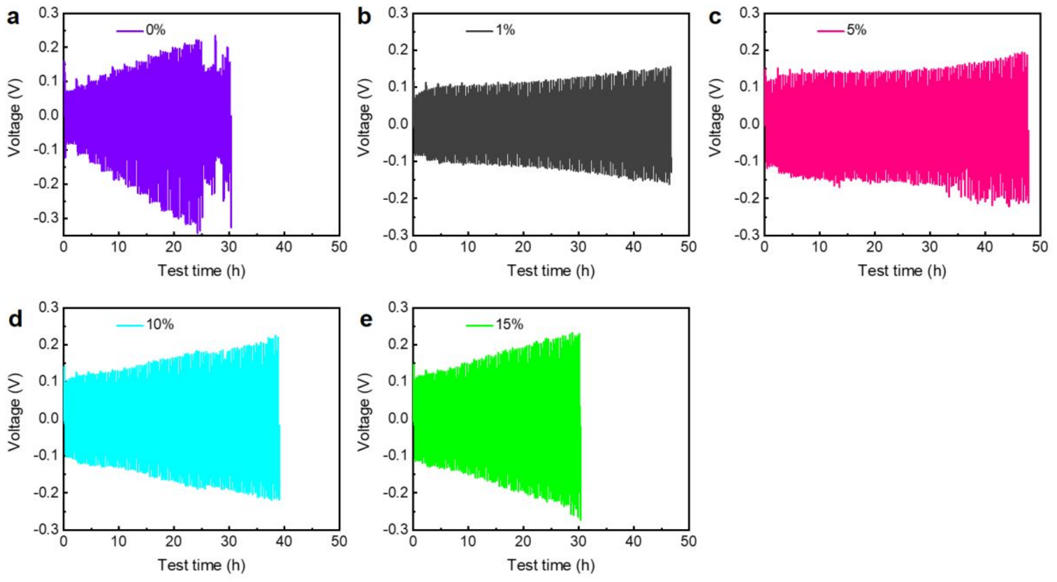

Figure S30. Rate performance of Zn||Zn symmetric cells at current densities from 0.5 mA cm^{-2} to 11 mA cm^{-2} in electrolytes with different percentages of EGME, 0% **a**, 1% **b**, 5% **c**, 10% **d** and 15% **e**.

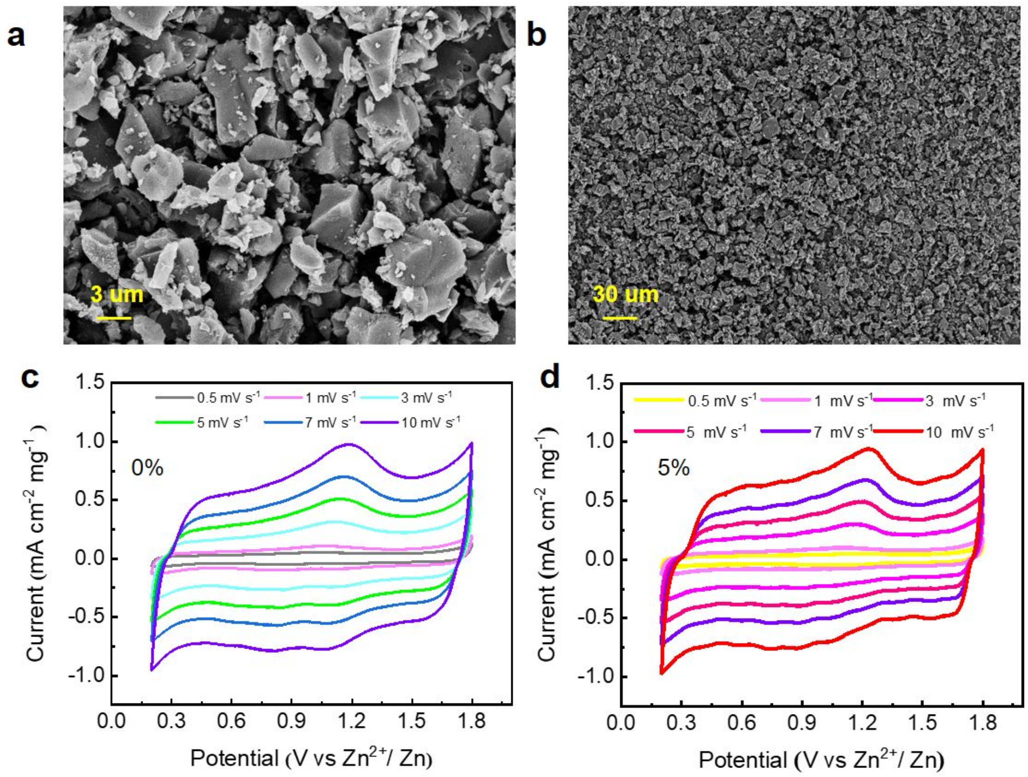

Figure S31. a, b, SEM image of AC; CV of AC in electrolytes without **c**/ with **d** EGME at different scan rate from 0.5 to 10 mV s^{-1}.

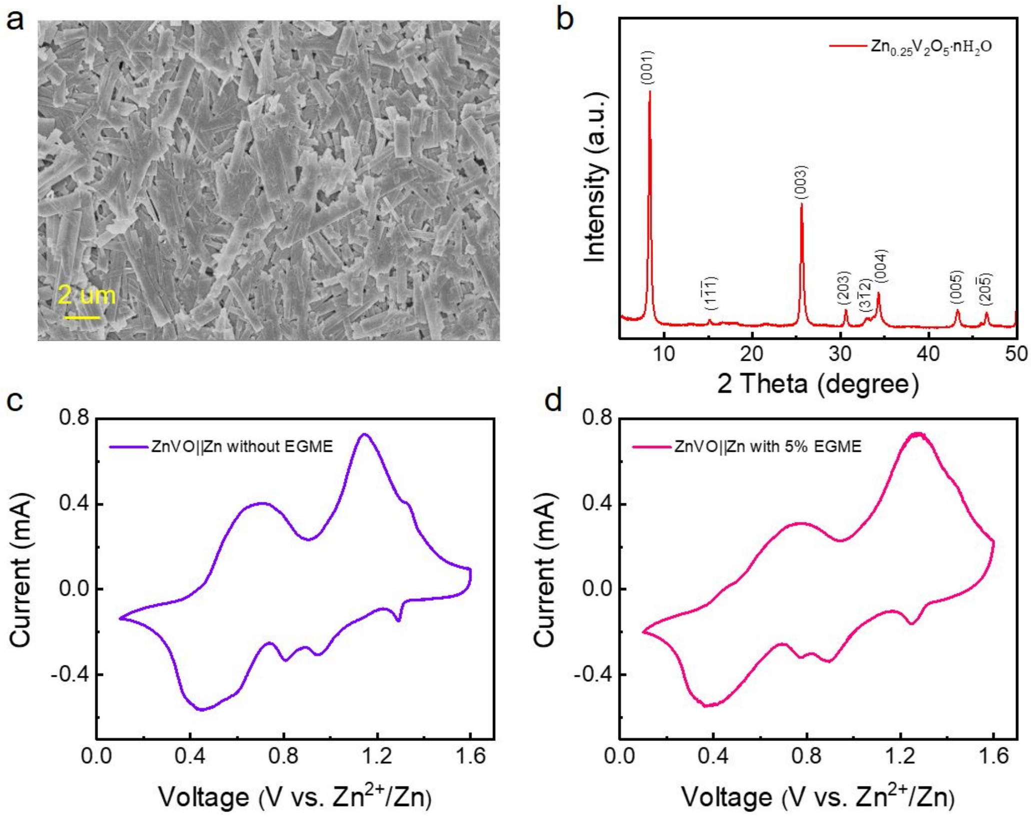

Figure S32. (a) SEM image and (b) XRD pattern of ZnVO material. The 2nd CV curves of

ZnVO||Zn cells without (c)/with (d) EGME at a scan rate of 0.5 mV s^{-1}.

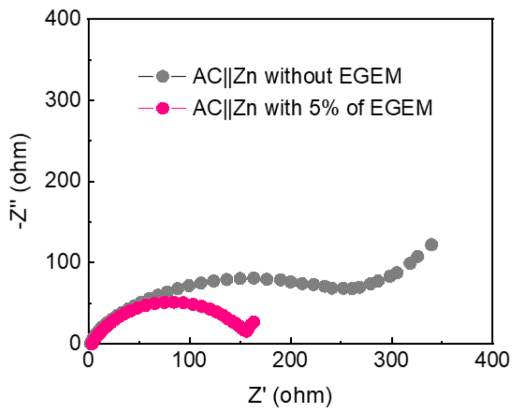

Figure S33. EIS tests for AC||Zn full cells with/without additive.

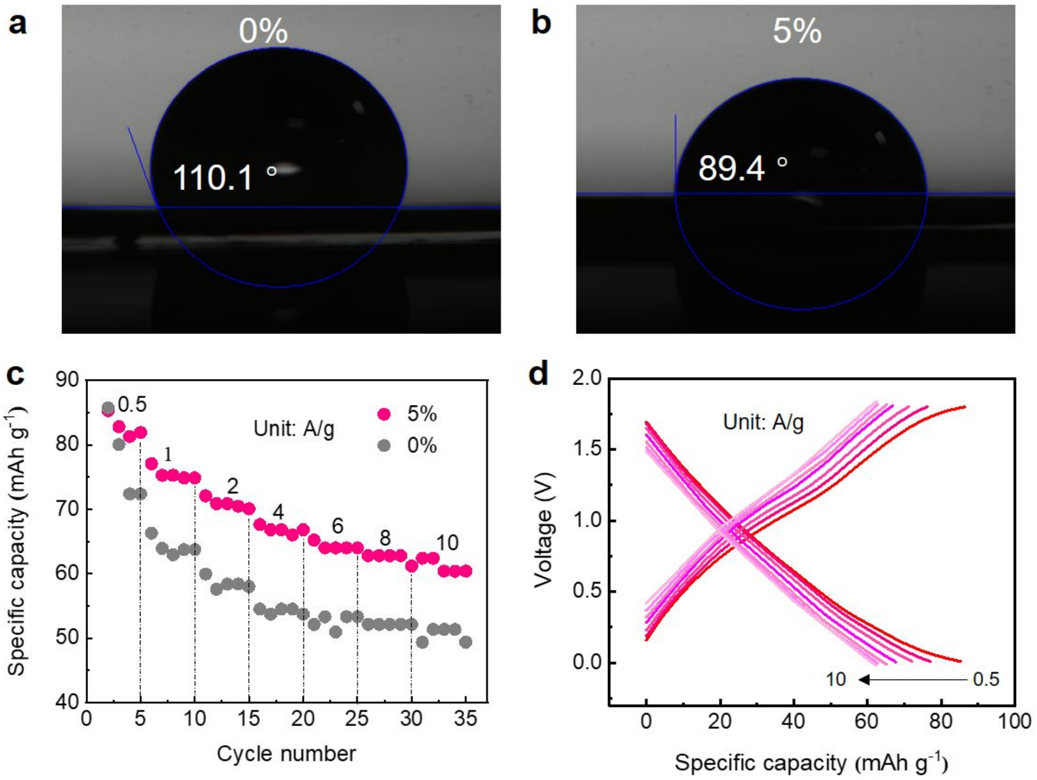

Figure S34. **a**, **b**, Wetting angles of electrolytes without/with EGME on AC electrode. **c**, Rate capability tests for AC||Zn full cells with/without additive. **d**, Galvanostatic charge and discharge curves of AC||Zn full coin cells at different rates in electrolytes with EGME.

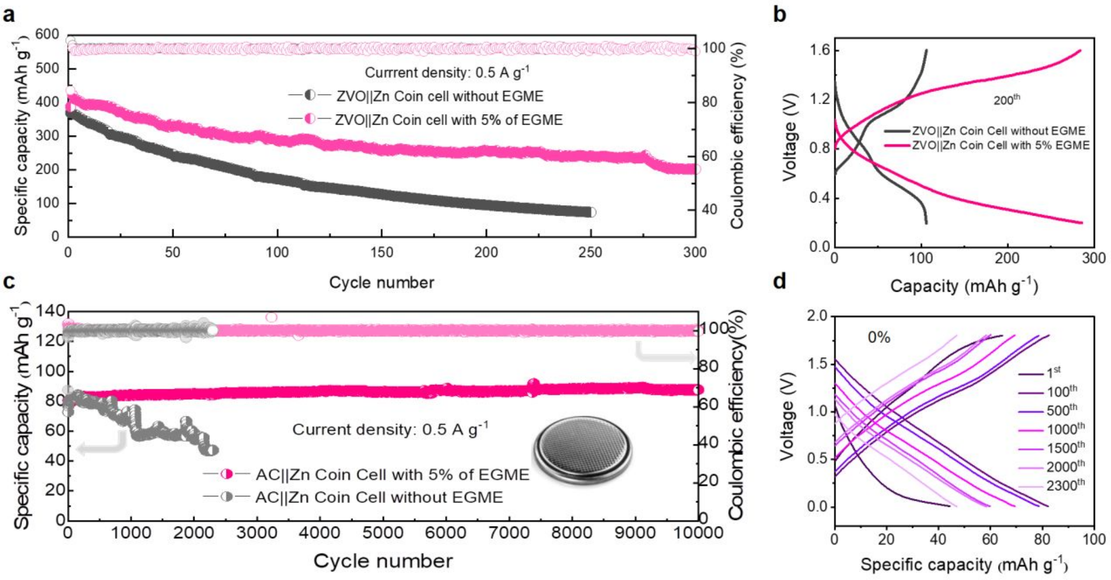

Figure S35. (a) Cycling stability and (b) corresponding galvanostatic charge/discharge curves of 200th cycles for ZnVO||Zn full coin cells without/with 5% of EGME. (c) Long-term cycling performance for AC||Zn full coin cells with/without the EGME additive at a current density of 0.5 A g^{-1}. (d) Corresponding galvanostatic charge and discharge curves of AC||Zn full coin cells long-term cycles without EGME.

Supplementary Tables

Table S1. EDS ratios from SEM of the sample without/with EGME.

0%			5%		
Element	Wt%	Wt% Sigma	Element	Wt%	Wt% Sigma
O	22.21	0.22	O	13.67	0.05
S	4.92	0.08	S	0.09	0.01
Cu	6.48	0.18	Cu	4.02	0.05
Zn	66.39	0.27	Zn	82.22	0.07
Total	100.00		Total	100.00	

Table S2. EDS ratios from TEM of the sample without/with EGME.

0%			5%		
Element	Wt%	Wt% Sigma	Element	Wt%	Wt% Sigma
O	15.26	0.15	O	22.78	0.15
S	6.58	0.09	S	0.08	0.03
Zn	78.16	0.17	Zn	77.14	0.15
Total	100.0		Total	100.00	

Table S3. The comparison of cyclability of Zn||Zn symmetric cells previously reported in literature with additives and those reported in this work at different current densities.

Zn-Zn Symmetric cells					
Electrolytes	Current density (mA cm^{-2})	Capacity (mAh cm^{-2})	Cycle number	Cycle Time(day)	Supplementary References
Mg^{2+}+$ZnSO_4$	1	0.25	600	13	[11] (AEM)
Na_4EDTA+$ZnSO_4$	5	2	2000	67	[12] (AEM)
Triethylmethyl ammonium+$ZnSO_4$ /$ZnCl_2$	1	0.5	2100	88	[13] (AEM)
NH_4OAc+$ZnSO_4$	1	1	1750	146	[14] (AEM)
TMBAC+$ZnSO_4$	1	2	1000	167	[15] (AEM)
N-methyl-2-pyrrolidone+$ZnSO_4$	1	1	550	46	[16] (AEM)
Amino acid+$ZnSO_4$	5	4	2200	147	[17] (AFM)
Triethyl phosphate+$Zn(OTf)_2$	1	1	750	63	[18] (AFM)
SeO_2+$ZnSO_4$	2	2	1050	88	[19] (AFM)
Propylene glycol+$ZnSO_4$	2	2	500	42	[20] (AFM)
Silk Peptide+$ZnSO_4$	1	1	1500	125	[21] (AFM)
Ch^{+}+$ZnSO_4$/$Zn(CH_3COO)_2$/$ZnCl_2$	1	1	1000	83	[22] (AFM)
$Zn(TFSI)_2$+LiTFSI	0.2	0.068	500	15	[23](Nat. Mater)
0.02M KFSI+2M $Zn(OTF)_2$	0.5	0.25	6000	250	[24](Nat. Nanotech)
$ZnCl_2$+DMSO	0.5	0.5	1000	83	[25] (JACS)
3M $Zn(CF_3SO_3)_2$	0.1	0.1	400	34	[26] (JACS)
$ZnSO_4$	2	2	140	12	[27](Nat. Commun)
$ZnSO_4$	0.5	0.5	1500	125	[28](Angew. Chem. Int. Ed.)
4M $Zn(BF_4)_2$ +2mM $Al(OTf)_3$)	1	1	1800	150	[29](Angew. Chem. Int. Ed.)
$ZnSO_4$+Glucose	5	5	150	13	[30](Angew. Chem. Int. Ed.)
$Zn(OTF)_2$+$Zn(NO_3)_2$	0.5	0.5	60	5	[31](Angew. Chem. Int. Ed.)
$ZnSO_4$	5	0.5	2000	17	[32](Angew. Chem. Int. Ed.)
$ZnSO_4$	0.2	0.2	400	33	[33](Angew. Chem. Int. Ed.)
ZnF_2+$Zn_3(PO_4)_2$	0.5	0.5	350	30	[34](Angew. Chem. Int. Ed.)
$ZnSO_4$	2	2	1100	92	[35](Adv. Mater)
$ZnSO_4$	2	1	2400	100	[36](Adv. Mater)
$Zn(TfO)_2$+PA	0.5	0.25	8000	333	[37](Energy Environ. Sci.)
$ZnSO_4$+0.1 M $MnSO_4$	5	1.25	1000	20	[38](Energy Environ. Sci.)
$ZnSO_4$	2	2	350	30	[39](Adv. Sci.)
$ZnSO_4$+$MnSO_4$	0.25	0.05	800	13	[40] (AEM)
$ZnSO_4$+$MnSO_4$	10	10	200	17	[41] (AEM)
$ZnSO_4$	1	1	25	2	[42] (Joule)
$Zn(CF_3SO_3)_2$+2vol % Et_2O	1	1	90	7	[43](Nano Energy)
$ZnSO_4$+68vol% EG	0.5	0.5	1334	111	[44](Nano Energy)
30M $ZnCl_2$	0.2	1/30	200	3	[45](Chem. Commun)
**$ZnSO_4$+EGME**	**1**	**0.5**	**8900**	**366**	**This work**

**4**	**0.5**	**3800**	**40**
**6**	**1.5**	**1600**	**33**
**12**	**3**	**200**	**4**

Supplementary References

1. Kundu, D., Adams, B. D., Du, V., Vajargah, S. H. & Nazar, L. F. A high-capacity and long-life aqueous rechargeable zinc battery using a metal oxide intercalation cathode. *Nat Energy* **1**, 16119 (2016).
2. N. Dubouis, A. Serva, E. S. The Fate of Water at the Electrochemical Interfaces: Electrochemical Behavior of Free Water Versus Coordinating Water. *J. Phys. Chem. Lett.* **9**, 6683– 6688 (2018).
3. Zheng, J. *et al.* Spontaneous and field-induced crystallographic reorientation of metal electrodeposits at battery anodes. *Sci. Adv.* **6**, eabb1122 (2020).
4. Cheng, X., Zhang, R., Zhao, C. & Zhang, Q. Toward Safe Lithium Metal Anode in Rechargeable Batteries : A Review. *Chem. Rev.* **117**, 10403 (2017).
5. Wang, D. *et al.* Insight on Organic Molecules in Aqueous Zn-Ion Batteries with an Emphasis on the Zn Anode Regulation. *Adv. Energy Mater.* **5–19**, 2102707 (2022).
6. Kashchiev, D. On the relation between nucleation work , nucleus size , and nucleation rate. *J. Chem. Phys.* **76**, 5098– 5102 (1982).
7. Oxtoby, D. W. A general relation between the nucleation work and the size of the nucleus in multicomponent nucleation. *J. Chem. Phys.* **100**, 7665 (1994).
8. Pei, A., Zheng, G., Shi, F., Li, Y. & Cui, Y. Nanoscale Nucleation and Growth of Electrodeposited Lithium Metal. *Nano Lett* **17**, 1132−1139 (2017).
9. Li, Q., Chen, A., Wang, D. & Pei, Z. “Soft Shorts” Hidden in Zinc Metal Anode Research. *Joule* **6**, 269–279 (2022).
10. Chuai, M. *et al.* Theory-Driven Design of a Cationic Accelerator for High-Performance Electrolytic MnO2 – Zn Batteries. *Adv.Mater.* **34**, 2203249 (2022).
11. Wang, P. *et al.* Mechanistic Insights of Mg 2 + -Electrolyte Additive for High-Energy and Long-Life Zinc-Ion Hybrid Capacitors. *Adv. Energy Mater.* **11**, 2101158 (2021).
12. Zhang, S. *et al.* Dual-Function Electrolyte Additive for Highly Reversible Zn Anode. *Adv. Energy Mater.* **11**, 2102010 (2021).
13. Yao, R. *et al.* A Versatile Cation Additive Enabled Highly Reversible Zinc Metal Anode. *Adv. Energy Mater* **12**, 2102780 (2022).
14. Han, D. *et al.* A Self-Regulated Interface toward Highly Reversible Aqueous Zinc Batteries. *Adv. Energy Mater* **12**, 2102982 (2022).
15. Guan, K. *et al.* Anti-Corrosion for Reversible Zinc Anode via a Hydrophobic Interface in Aqueous Zinc Batteries. *Adv. Energy Mater* **12**, 2103557 (2022).

16. Li, T. C. *et al.* A Universal Additive Strategy to Reshape Electrolyte Solvation Structure toward Reversible Zn Storage. *Adv. Energy Mater.* **12**, 2103231 (2022).

17. Lu, H. *et al.* Amino Acid-Induced Interface Charge Engineering Enables Highly Reversible Zn Anode. *Adv. Funct. Mater.* **31**, 2103514 (2021).

18. Liu, S. *et al.* Tuning the Electrolyte Solvation Structure to Suppress Cathode Dissolution , Water Reactivity , and Zn Dendrite Growth in Zinc-Ion Batteries. *Adv. Funct. Mater* **31**, 2104281 (2021).

19. Huang, C. *et al.* Self-Healing SeO 2 Additives Enable Zinc Metal Reversibility in Aqueous ZnSO 4 Electrolytes. *Adv. Funct. Mater.* **32**, 2112091 (2022).

20. Shang, Y. *et al.* Long-Life Zn Anode Enabled by Low Volume Concentration of a Benign Electrolyte Additive. *Adv. Funct. Mater.* **32**, 2200606 (2022).

21. Wang, B. *et al.* Synergistic Solvation and Interface Regulations of Eco-Friendly Silk Peptide Additive Enabling Stable Aqueous Zinc-Ion Batteries. *Adv. Funct. Mater.* **2112693**, 32 (2022).

22. Nie, X. *et al.* Cholinium Cations Enable Highly Compact and Dendrite-Free Zn Metal Anodes in Aqueous Electrolytes. *Adv. Funct. Mater* 2203905 (2022).

23. Wang, F. *et al.* Highly reversible zinc metal anode for aqueous batteries. *Nat. Mater.* **17**, 543–550 (2018).

24. Cao, L. *et al.* Fluorinated interphase enables reversible aqueous zinc battery chemistries. *Nat. Nanotechnol.* **16**, 902–910 (2021).

25. Cao, L. *et al.* Solvation Structure Design for Aqueous Zn Metal Batteries. *J. Am. Chem. Soc.* **142**, 21404−21409 (2020).

26. Zhang, N. *et al.* Cation-Deficient Spinel ZnMn2O4 Cathode in Zn(CF3SO3)2 Electrolyte for Rechargeable Aqueous Zn-Ion Battery. *J. Am. Chem. Soc.* **138**, 12894−12901 (2016).

27. Zhang, Q. *et al.* Revealing the role of crystal orientation of protective layers for stable zinc anode. *Nat. Commun.* **11**, 3961 (2020).

28. Yang, H. *et al.* Zinc Batteries Hot Paper Constructing a Super-Saturated Electrolyte Front Surface for Stable Rechargeable Aqueous Zinc Batteries. *Angew. Chem. Int. Ed.* **59**, 1–6 (2020).

29. Longtao Ma, Shengmei Chen, Xinliang Li, Ao Chen, Binbin Dong, and C. Z. Liquid-free all-solid-state Zn batteries and encapsulation-free flexible batteries enabled by in-situ constructed polymer electrolyte. *Angew.Chem.Int.Ed.* **59**, 3836–23844 (2020).

30. Sun, P. *et al.* Simultaneous Regulation on Solvation Shell and Electrode Interface for Dendrite-Free Zn Ion Batteries Achieved by a Low-Cost Glucose Additive Research Articles. *Angew. Chem. Int. Ed.* **60**, 18247–18255 (2021).

31. Li, D., Cao, L., Deng, T., Liu, S. & Wang, C. Design of a Solid Electrolyte Interphase for Aqueous Zn Batteries. *Angew. Chem. Int. Ed.* **60**, 13035–13041 (2021).

32. Cui, Y. *et al.* An Interface-Bridged Organic–Inorganic Layer that Suppresses Dendrite Formation and Side Reactionsfor Ultra-Long-Life Aqueous Zinc Metal Anodes. *Angew.Chem.Int.Ed.* **59**, 16594–16601 (2020).

33. Zhang, N. *et al.* Direct Self-Assembly of MXene on Zn Anodes for Dendrite-Free Aqueous Zinc-Ion Batteries. *Angew. Chem. Int. Ed.* **60**, 2861–2865 (2021).

34. Cao, L., Li, D., Deng, T., Li, Q. & Wang, C. Hydrophobic Organic-Electrolyte-Protected Zinc Anodes for Aqueous Zinc Batteries. *Angew. Chem. Int. Ed.* **59**, 19292–19296 (2020).

35. Hao, J. *et al.* An In-Depth Study of Zn Metal Surface Chemistry for Advanced Aqueous Zn-Ion Batteries. *Adv. Mater* 2003021 (2020).

36. Yang, Q. *et al.* Hydrogen-Substituted Graphdiyne Ion Tunnels Directing Concentration Redistribution for Commercial-Grade Dendrite-Free Zinc Anodes. *Adv. Mater.* **32**, 2001755 (2020).

37. Zhao, Z. *et al.* Long-life and deeply rechargeable aqueous Zn anodes enabled by a multifunctional brightener-inspired interphase. *Energy Environ. Sci.* **12**, 1938 (2019).

38. Xie, X. *et al.* Manipulating the ion-transfer kinetics and interface stability for high-performance zinc metal anodes. *Energy Environ. Sci.* **13**, 503–510 (2020).

39. Liu, X. *et al.* Zeolitic Imidazolate Frameworks as Zn2+ Modulation Layers to Enable Dendrite-Free Zn Anodes. *Adv. Sci.* 2002173 (2020).

40. Kang, L. *et al.* Nanoporous CaCO3 Coatings Enabled Uniform Zn Stripping/ Plating for Long-Life Zinc Rechargeable Aqueous Batteries. *Adv. Energy Mater.* **8**, 1801090 (2018).

41. Yuksel, R., Buyukcakir, O., Seong, W. K. & Ruoff, R. S. Metal-Organic Framework Integrated Anodes for Aqueous Zinc-Ion Batteries. *Adv. Energy Mater.* 1904215 (2020).

42. Wang, Z. *et al.* A Metal-Organic Framework Host for Highly Reversible Dendrite-free Zinc Metal Anodes. *Joule* **3**, 1289–1300 (2019).

43. Xu, W. *et al.* Diethyl ether as self-healing electrolyte additive enabled long-life rechargeable aqueous zinc ion batteries. *Nano Energy* **62**, 275–281 (2019).

44. Xu, W. *et al.* Diethyl ether as self-healing electrolyte additive enabled long-life rechargeable aqueous zinc ion batteries. *Nano Energy* **62**, 275–281 (2019).

45. C. Zhang, J. Holoubek, X. Wu, A. Daniyar, L. Zhu, C. C. & D. P. Leonard, I. A. Rodriguez-Perez, J.-X. Jiang, C. Fang, X. J. ZnCl2 Water-in-Salt Electrolyte for Reversible Zn Metal Anode. *Chem. Commun.* **54**, 14097 (2018).
